# Dual‐Function Ceramic Pigments for Energy‐Efficient and Secure Autonomous Vehicles

**DOI:** 10.1002/advs.202503901

**Published:** 2025-04-30

**Authors:** Ik Hoon Jeong, Yong‐Jun Seo, Jum Soo Hwang, Geun Hyeong Kim, Yeong Jae Kim, Gil Ju Lee

**Affiliations:** ^1^ School of Electrical and Electronics Engineering Pusan National University Busandaehak‐ro 63, Geumjeong‐gu Busan 46241 Republic of Korea; ^2^ Ceramic Total Solution Center Korea Institute of Ceramic Engineering of Technology Gyeongchung‐daero 3321, Sindun‐myeon, Icheon‐si Icheon Gyeonggi‐do 17303 Republic of Korea; ^3^ DEERS i CO., LTD Cheongbuksandan‐ro 76, Cheongbuk‐myeon Pyeongtaek‐si Gyeonggi‐do 17792 South Korea

**Keywords:** anti‐counterfeiting, ceramic pigment, doping, LiDAR detection, MIE scattering, near‐infrared reflectance, radiative cooling

## Abstract

The global automobile market continues to demonstrate robust growth and innovation potential. Vehicles have evolved into complex, technology‐intensive platforms, and autonomous vehicles are anticipated to become commonplace in the near future. However, key challenges persist, such as managing excessive energy consumption and ensuring precise object detection. Under intense sunlight, elevated in‐vehicle temperatures lead to higher demand for cooling systems, ultimately increasing energy use. Furthermore, autonomous driving necessitates accurate detection of surrounding vehicles. To address these issues, doped ceramic pigments with diverse colors are developed, exhibiting enhanced near‐infrared (NIR) reflectance across the solar spectrum, thereby achieving a radiative cooling (RC) effect. In addition, to improve detectability by LiDAR, scattering analyses are performed to investigate how ceramic particles scatter incident light in various directions. The results indicate that although the scattering is not strictly retro‐reflective, it strongly redirects the incident wave back toward its source, which is advantageous for LiDAR‐based object detection. Consequently, a bi‐function ceramic pigment (BFCP) is fabricated with both superior RC performance and heightened LiDAR detectability compared to commercial pigments. Moreover, integrating BFCP with commercial pigments enables anti‐counterfeiting strategies, as letters or patterns invisible to the naked eye can be visualized under IR‐mode cameras.

## Graphical abstract

This study developed and tested a vehicle pigment designed for enhanced LiDAR detectability and radiative cooling. By incorporating ceramic materials and employing numerical simulations, the pigment is designed to maximize its effectiveness. Consequently, this pigment enhances the safety of autonomous driving while mitigating interior temperature increases during the summer, and a range of applications is feasible, including anti‐counterfeiting.

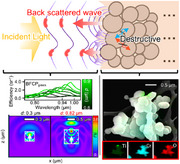

## 1 Introduction

The automobile industry, initially developed as a straightforward means of transportation, has evolved into a sophisticated arena for performing numerous tasks on the move.^[^ ^1^, ^2^ ^]^ In response to growing concerns about climate change, significant efforts are being made to develop advanced green automotive technologies, such as electric vehicles.^[^ ^3^, ^4^ ^]^ Yet, under strong sunlight, in‐vehicle temperatures can rise substantially, leading to an increased dependence on conventional air‐conditioning systems and, consequently, greater energy consumption.^[^ ^5^ ^]^

Radiative cooling (RC) technology offers a promising solution to mitigate this excessive energy use. By reflecting a major portion of solar irradiance (primarily 0.3–2.5 µm) and emitting thermal radiation through the atmospheric window in the long‐wave infrared (LWIR, 8–13 µm) region, RC can maintain lower surface temperatures even under direct sunlight.^[^ ^6^, ^7^, ^8^, ^9^, ^10^ ^]^ Achieving near‐unity solar reflectance (*R̅_solar_* ≈ 1.0) is critical; even minimal solar absorption can significantly increase surface temperatures despite high thermal emission.

Meanwhile, autonomous driving technology, along with electric vehicle advancements, is emerging as a key innovation in the automotive industry, given its substantial potential to enhance both convenience and safety. Autonomous driving systems crucially depend on robust object detection, typically using light detection and ranging (LiDAR).^[^ ^11^, ^12^ ^]^ LiDAR operates by emitting pulses of near‐infrared (NIR) light (e.g., 904–940 nm) and measuring the time of flight to capture the position of objects.^[^ ^13^ ^]^ For reliable detection, maximizing the return signal—regardless of incident angle—is essential.

However, currently, available commercial pigments (CMPs) are primarily designed for aesthetic variety, rendering it difficult to enhance R̅_solar_ in the visible region (e.g., 0.38–0.75 µm). As a result, these pigments typically exhibit relatively low R̅_solar_ (i.e., < 0.31), making them suboptimal for RC applications. In addition, LiDAR reflectance (R̅_LiDAR_) in the NIR range (e.g., 0.75–2.5 µm) is often low and varies significantly with the angle of incidence, hindering effective detection.

In this work, we introduce bi‐function ceramic pigments (BFCPs) produced via simple thermal calcination of metal oxide micro–nano particles. By doping ceramic materials with titanium dioxide (TiO₂), manganese dioxide (MnO₂), and tantalum pentoxide (Ta₂O₅), we enhance NIR reflectance within the solar spectrum, thereby increasing R̅_solar_ for RC.^[^ ^14^, ^15^, ^16^, ^17^ ^]^ To ensure robust LiDAR detection irrespective of viewing angle, we numerically analyzed Mie scattering to identify particle diameters that maximize backscattering in the direction of incidence within the LiDAR spectrum range (e.g., 904–940 nm).^[^ ^18^ ^]^ While not strictly retro‐reflective, these doped ceramic particles strongly redirect the incident beam back toward its source, benefiting LiDAR detection. Consequently, BFCPs offer both RC effectiveness and superior LiDAR detectability. Additionally, BFCPs, in conjunction with commercial pigments, enable anti‐counterfeiting features by displaying IR‐visible patterns that remain invisible to the naked eye (Figure S18, Supporting Information).

Through these dual functionalities—energy‐saving radiative cooling and reliable LiDAR detection—our BFCPs promise a significant contribution to next‐generation green automotive technologies.

## 2 Result

### 2.1 Design of BFCP

**Figure 1:**
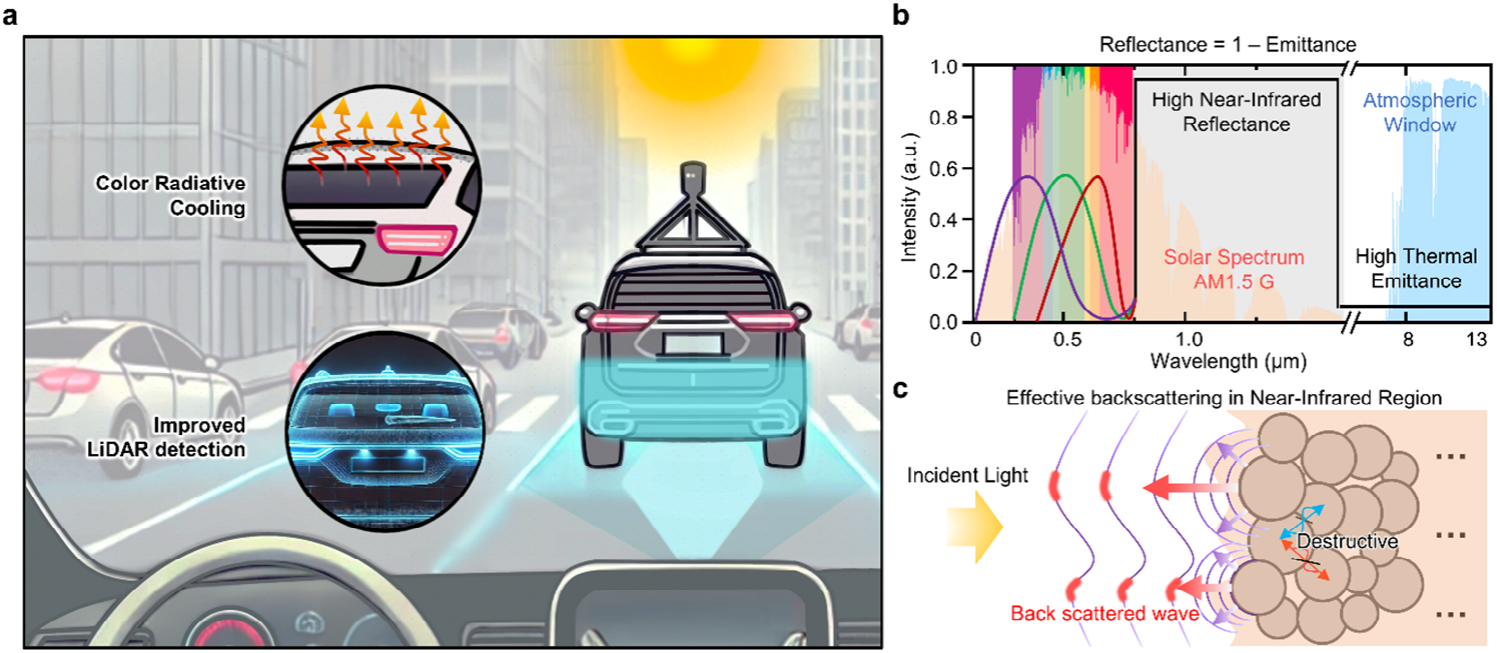
Schematic of bi‐functional ceramic pigment (BFCP). a) Schematic of the effects of BFCP. The vehicle coated with BFCP exhibits dual functionality; radiative cooling (RC) and improved LiDAR detection. b) Graph depicting the ideal BFCP for RC effect. The optimal BFCP, available in various colors exhibits near‐unit reflectance in the near‐infrared region (NIR, 0.75–2.5 µm) and the high thermal emittance in the long‐wave infrared region (LWIR, 8–13 µm). c) Schematic of effective backscattering by BFCP. The backscattered wave is effectively generated from BFCP containing optimally sized particles designed for enhanced backscattering.

**Figure 2:**
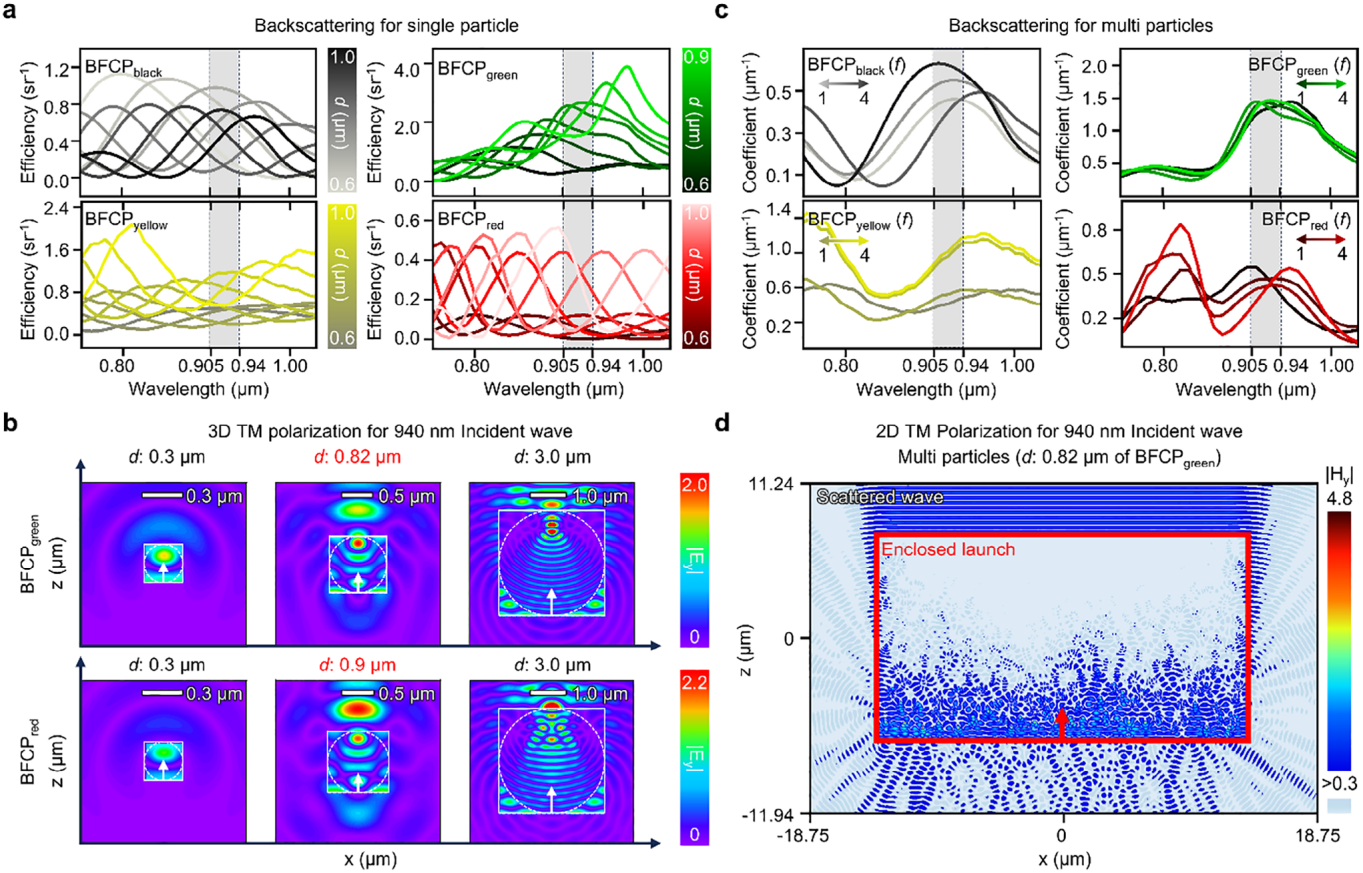
Design of BFCP. a) Graph depicting the backscattering efficiency of single particles. Due to the dependence of peak backscattering shifts on particle diameter, we can estimate the optimal diameter for each BFCP (e.g., BFCP_black_ = 0.7 µm, BFCP_green_ = 0.82 µm, BFCP_yellow_ = 0.9 µm, and BFCP_red_ = 0.9 µm). b) Cross‐sectional electric field maps of single BFCP particles. For a 940 nm incident wave (*λ*), when the diameter (*d*) of BFCP_green_ or BFCP_red_ is comparable to the incident wave (i.e.*, d* ∼ *λ*), the backscattered electric field is more effective than in other cases (i.e.*, d*≪ *λ* and *d*≫ *λ*). The results for BFCP_black_ and BFCP_yellow_ are provided in Figure S4 (Supporting Information). c) Graph of the backscattering coefficient of multi particles. Based on the volume concentration (*f*) presented in Table 1, the effective scattering coefficient of multi‐particles can be estimated. d) Cross‐sectional electric field maps of multiple BFCP particles. For a 940 nm incident wave (*λ*), when BFCP_green_ is composed of optimally sized particles (e.g.*, d* = 0.82 µm), the cross‐sectional map illustrates a pronounced backscattered electric field.

As illustrated in **Figure** **1**a, our results show that the engineered coating simultaneously achieves a highly efficient passive RC effect and strong LiDAR detection capability. Two primary objectives guide the design of this coating: 1) boosting NIR reflectance and thermal emittance while preserving a stable color range in the visible spectrum as presented in Figure 1b, and 2) enhancing the effective backscattering by micro–nano particles, defined as the intensity of scattered waves returning toward the incident beam back toward its source as depicted in Figure 1c.

To address the first objective, the BFCP is fabricated using metal oxide ceramic materials composed of ionic bonds with oxygen. These materials exhibit distinct colors and strong thermal emissions in the LWIR region, owing to the vibrational response of their ionic bonds.^[^ ^19^, ^20^ ^]^ To further improve NIR reflectance, based on the ceramic‐based doping approach for optical property enhancement described in Method S1 (Supporting Information), ceramic materials of various colors are doped with manganese (Mn^4+^), titanium (Ti^4+^) and tantalum (Ta^5+^) ions.^[^ ^14^, ^15^, ^16^, ^17^ ^]^ Due to the properties of ceramic materials doped with the ions, following the definition of the extinction coefficient, incident light is significantly influenced by the material rather than being easily transmitted.^[^ ^21^, ^22^ ^]^ This is attributed to the substantial refractive index contrast within the NIR range between the material (*n*≥ 2.5) and its surrounding media, such as polydimethylsiloxane (PDMS, *n* = 1.4). Furthermore, the materials exhibit a low absorption coefficient (i.e.*, k* < 0.1). Consequently, Light that is not transmitted due to the material's influence undergoes stronger scattering than absorption in the NIR range, leading to superior NIR reflectance compared to CMP.^[^ ^21^ ^]^ To fulfill the second objective, the design process mainly targets Mie scattering phenomena in particle‐induced scattering processes.^[^ ^23^, ^24^ ^]^ When light propagating through a medium (PDMS) encounters BFCP particles, the resulting scattering is governed by the particles’ intrinsic optical properties. Among various scattering mechanisms, Mie scattering—occurring when the wavelength (*λ*) of the light is on the order of the particle diameter (*d*)—exhibits an enhanced tendency for backscattering relative to other scattering types.^[^ ^24^ ^]^ Consequently, for the LiDAR detection range (e.g., 904–940 nm), optimizing the particle size distribution is critical for achieving strong LiDAR return signals (Figure 1c). Through this strategy, the BFCP is designed to yield improved backscattering efficiency, thereby enhancing LiDAR detection performance.

### 2.2 Production of BFCP Based on Numerical Simulation

When light interacts with a particle, the resulting scattered light is emitted in various directions due to interactions between the particle and incident photons.^[^ ^21^ ^]^ To characterize how the direction of scattered waves changes with different scattering conditions, we computed the phase function of the scattered light based on the Mie theory and scattered radiation methodology described in Method S3 (Supporting Information).^[^ ^21^, ^23^, ^24^ ^]^ The phase function indicates that the scattering profile depends on the underlying mechanism—Rayleigh, Mie, or Geometric scattering as shown in Figure S1 (Supporting Information). From this analysis, Mie scattering is expected to exhibit a stronger tendency for efficient backscattering than other types of scattering.^[^ ^24^ ^]^ Backscattering efficiency, defined as the fraction of incident light redirected as retro‐reflection within the backward‐scattering angle range, was calculated as a function of particle diameter (*d*) to identify the optimal conditions for maximizing backscattering following the backscattering procedure explained in Method S4 (Supporting Information).^[^ ^21^, ^25^ ^]^ Consequently, according to **Figure** **2**a, the peak backscattering wavelength depends on the diameter of each BFCP. These results show that each BFCP—distinguished by color—has an optimal particle diameter for LiDAR backscattering, which is further visualized by cross‐sectional electric field maps as shown in Figure 2b and Figure S4 (Supporting Information). Notably, among various scattering mechanisms, Mie scattering demonstrates the strongest propensity for back‐scattered electric fields, and BFCP particles at their optimal diameters (*d*) significantly enhance backscattering.^[^ ^26^, ^27^ ^]^

Because BFCP consists of multiple particles with randomly distributed sizes and positions, achieving high numerical accuracy is challenging.^[^ ^23^, ^24^, ^28^ ^]^ To address these complexities, we adopted two primary assumptions (details in Method S3, Supporting Information): 1) scattering is independently generated by individual particles, and 2) the particles are uniformly dispersed in the base material (e.g., PDMS) at a specific particle volume concentration (*f*). By calculating the backscattering within this heterogeneous PDMS matrix for three different particle diameters within the LiDAR detection range over various volume concentrations in **Table** **1**, using the scattered radiation methodology in Method S4 (Supporting Information), we identified the most efficient BFCP configurations (Figure 2c). In these optimized configurations, Figure 2d and Figure S5 (Supporting Information) illustrate that the cross‐section magnetic field distribution reveals a pronounced back‐scattered magnetic field.^[^ ^26^, ^27^ ^]^

**Table 1:** BFCP configuration with volume concentration (*f*) of three optimal particle sizes. For the calculation of the effective scattering coefficient, the volume ratio between PDMS and particles is set to 1:0.3.

BFCP_black_		*d* (µm)	*f*	*d* (µm)	*f*	*d* (µm)	*f*	BFCP_green_		*d* (µm)	*f*	*d* (µm)	*f*	*d* (µm)	*f*
	*f1*	0.7	0.1	0.75	0.1	0.95	0.1		*f1*	0.8	0.1	0.82	0.1	0.85	0.1
	*f2*	0.7	0.2	0.75	0.1				*f2*	0.8	0.1	0.82	0.2		
	*f3*	0.75	0.3						*f3*	0.8	0.3				
	*f4*	0.7	0.3						*f4*	0.82	0.3				
BFCP_yellow_	*f1*	0.9	0.1	0.95	0.1	1.0	0.1	BFCP_red_	*f1*	0.9	0.1	0.85	0.1	1.0	0.1
	*f2*	0.9	0.2	0.95	0.1				*f2*	0.9	0.2	1.0	0.1		
	*f3*	0.95	0.3						*f3*	0.9	0.2	0.85	0.1		
	*f4*	0.9	0.3						*f4*	0.9	0.3				

**Figure 3:**
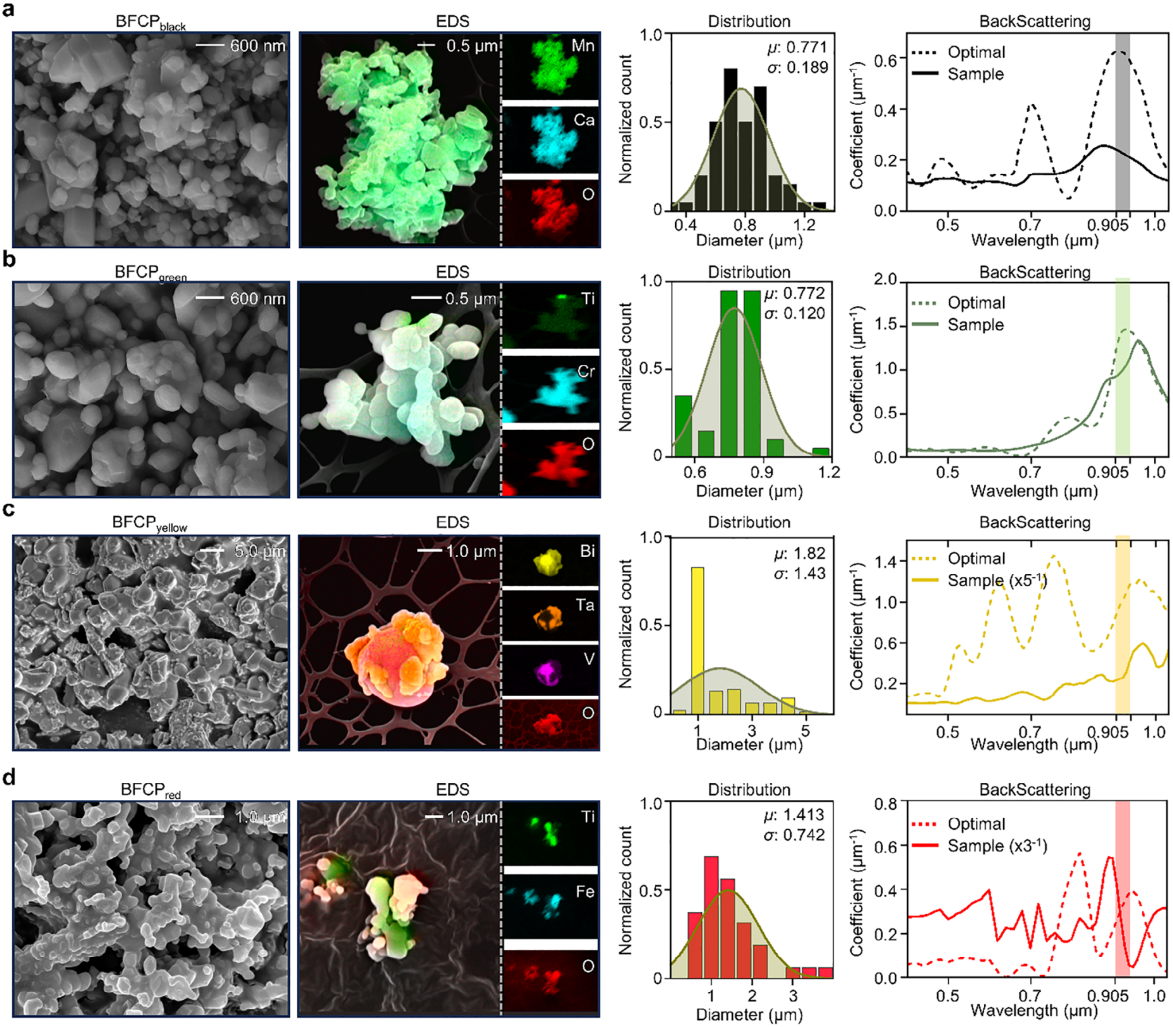
Analysis of BFCP sample. To examine the structural properties of the BFCP sample, the BFCP powder was first observed under magnification, followed by compositional analysis using Scanning Electron Microscope/Energy Dispersive Spectroscopy (SEM/EDS). By analyzing the particle size distribution with SEM images, the effective backscattering coefficient of each BFCP sample was calculated and compared with that of the optimal BFCP. a) Analysis of BFCP_black_. b) Analysis of BFCP_green_. c) Analysis of BFCP_yellow_. d) Analysis of BFCP_red_.

To compare the optimized BFCP configuration with actual BFCP samples synthesized by a thermal doping process (see Methods and Figure S3, Supporting Information), we analyzed the composition and size of the resulting ceramic powders using scanning electron microscopy (SEM), energy‐dispersive spectroscopy (EDS), and X‐ray diffraction (XRD; Figure S3, Supporting Information). Based on these measurements, we calculated the backscattering coefficient of each BFCP sample according to its volume concentrations (*f*) following the scattering in scattered radiation methodology described in Method S4 (Supporting Information). The black and green BFCP powders contained a high proportion of optimally sized particles and demonstrated backscattering properties resembling those of the optimized BFCP configuration (**Figure** **3**a,b; Figure S6, Supporting Information). Although the yellow and red samples also contained a substantial fraction of optimally sized particles, they included a higher proportion of larger particles, slightly reducing their overall backscattering coefficient while retaining a similar trend as described in Figure 3c,d, and Figure S6 (Supporting Information).

### 2.3 Optical Properties of BFCP

To fulfill the commercial and aesthetic demands of both consumers and the industry, samples with varying saturation levels were fabricated. To achieve different saturation levels, each BFCP (i.e., Black, Green, Yellow, and Red) was mixed with white pigment. To fabricate a BFCP with a pure white appearance, micro–nanomaterial (i.e., Al_2_O_3_, Figure S1, Supporting Information) was selected as the base component. Moreover, Figure S2 (Supporting Information) illustrates the design of an optical property enhancement approach based on ceramic doping.

**Figure 4:**
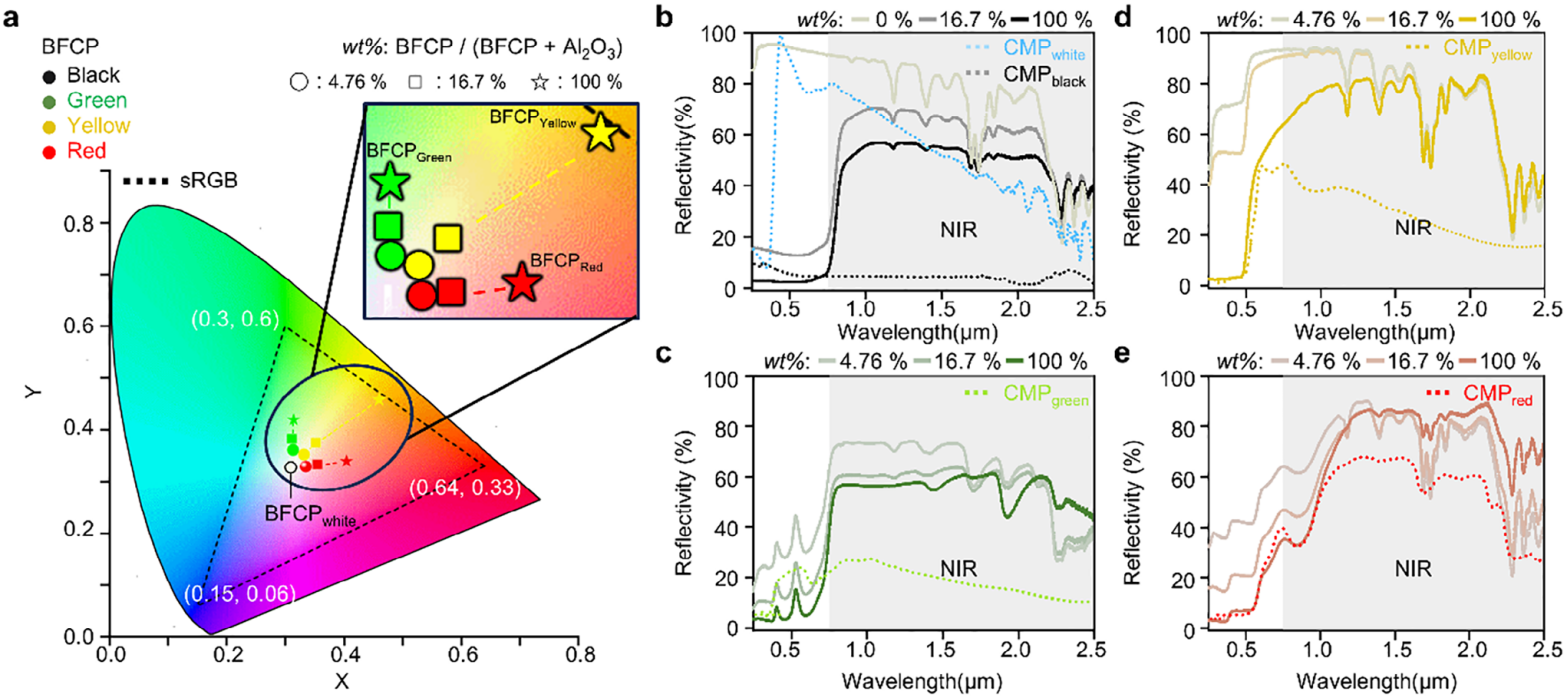
Spectral and optical properties of BFCP. a) Chromatic characteristic of BFCP in the color space. Using the tristimulus values (x, y, and z) of BFCP, coordinates (X, Y) in the CIE 1931 color space of chromaticity are derived. The result is that as the mass proportion of white pigment of BFCP (i.e., wt.%: BFCP/(BFCP + Al_2_O_3_), BFCP_white_: Al_2_O_3_) increases, the color saturation increases and shifts toward the white point. b) Graph of the optical characteristics of BFCP_black_. The optical properties were measured using a spectrophotometer in the visible range (e.g., 0.4–2.5 µm). BFCP_black_ with wt.% = 100% exhibits higher NIR reflectance than CMP_black_. As wt.% decreases, NIR reflectance is further enhanced. Additionally, BFCP with wt.% = 0% demonstrates improved NIR reflectance compared to CMP_white_. c) Graph of the optical characteristics of BFCP_green_. d) Graph of the optical characteristics of BFCP_yellow_. e) Graph of the optical characteristics of BFCP_red_.

After mixing colored BFCP with BFCP_white_ (i.e., Al_2_O_3_) at a specific weight ratio (i.e., wt.%), a film was subsequently fabricated (**Figure** **4**a; Tables S1–S3, Supporting Information). As shown in Figure S7 (Supporting Information), a decrease in wt.% corresponds to higher saturation. In the chromaticity diagram, which quantitatively represents human‐perceivable colors as points in the 2D color space, the sRGB reference is defined by three vertices: Red (X = 0.64, Y = 0.33), Green (X = 0.30, Y = 0.60), and Blue (X = 0.15, Y = 0.06). The triangular area connecting these points represents the sRGB color space.^[^ ^29^, ^30^, ^31^ ^]^ Plotting the BFCP samples shows that all data points lie within the sRGB boundary. Furthermore, as the wt.% of BFCP decreases, the color points shift toward the white point (X = 0.31, Y = 0.33), indicating higher saturation.

Subsequent reflectivity measurements (see Methods and Figure 4b–e) confirm that BFCP and CMP exhibit comparable reflectivity in the visible range. However, in the NIR range, BFCP shows the difference in reflectivity (%) was observed in the NIR range. Using reflectivity (%), reflectance, and thermal emittance were calculated as functions of wavelength following the definition of reflectance, and thermal emittance in the Methods section.^[^ ^10^, ^31^, ^32^ ^]^ As demonstrated in **Table** **2**, both BFCP and CMP exhibit high thermal emission (i.e., Δε ≤ 0.03), indicating efficient heat radiation. Additionally, there is no significant difference in reflectance within the visible region (i.e., $\Delta {\bar{R}}_{vis}\leq 0.1$); however, the difference in reflectance in the NIR range (i.e., $\Delta {\bar{R}}_{NIR}\leq 0.43$) is substantial. Consequently, the overall reflectance in the solar spectrum (i.e., $\Delta {\bar{R}}_{solar}\leq 0.23$) also exhibits a notable difference. Hence, BFCP is expected to be more effective in RC effect than CMP. Furthermore, a decrease in wt.% is observed to correlate with higher overall reflectance in the visible range, enhanced saturation, and improved reflectance in the NIR band (${\bar{R}}_{NIR}$).

**Table 2:** Optical performance: reflectance and emittance of BFCP and CMP.

Sample	Black		Green		Yellow		Red	
	CMP	BFCP	CMP	BFCP	CMP	BFCP	CMP	BFCP
${\bar{R}}_{\mathrm{VIS}}$ *_(0.4 ∼0.75_* *_µm)_*	0.05	0.05	0.21	0.11	0.27	0.37	0.16	0.17
${\bar{R}}_{\mathrm{NIR}}$ *_(0.75 ∼2.5_* *_µm)_*	0.04	0.47	0.24	0.57	0.37	0.74	0.50	0.60
${\bar{R}}_{\mathrm{solar}}$ *_(0.25∼2.5_* *_µm)_*	0.05	0.25	0.22	0.32	0.30	0.53	0.31	0.37
$\bar{\varepsilon }$ *_(8 ∼13_* *_µm)_*	0.94	0.96	0.97	0.96	0.92	0.95	0.96	0.95

### 2.4 Radiative Cooling (RC) Test

**Figure 5:**
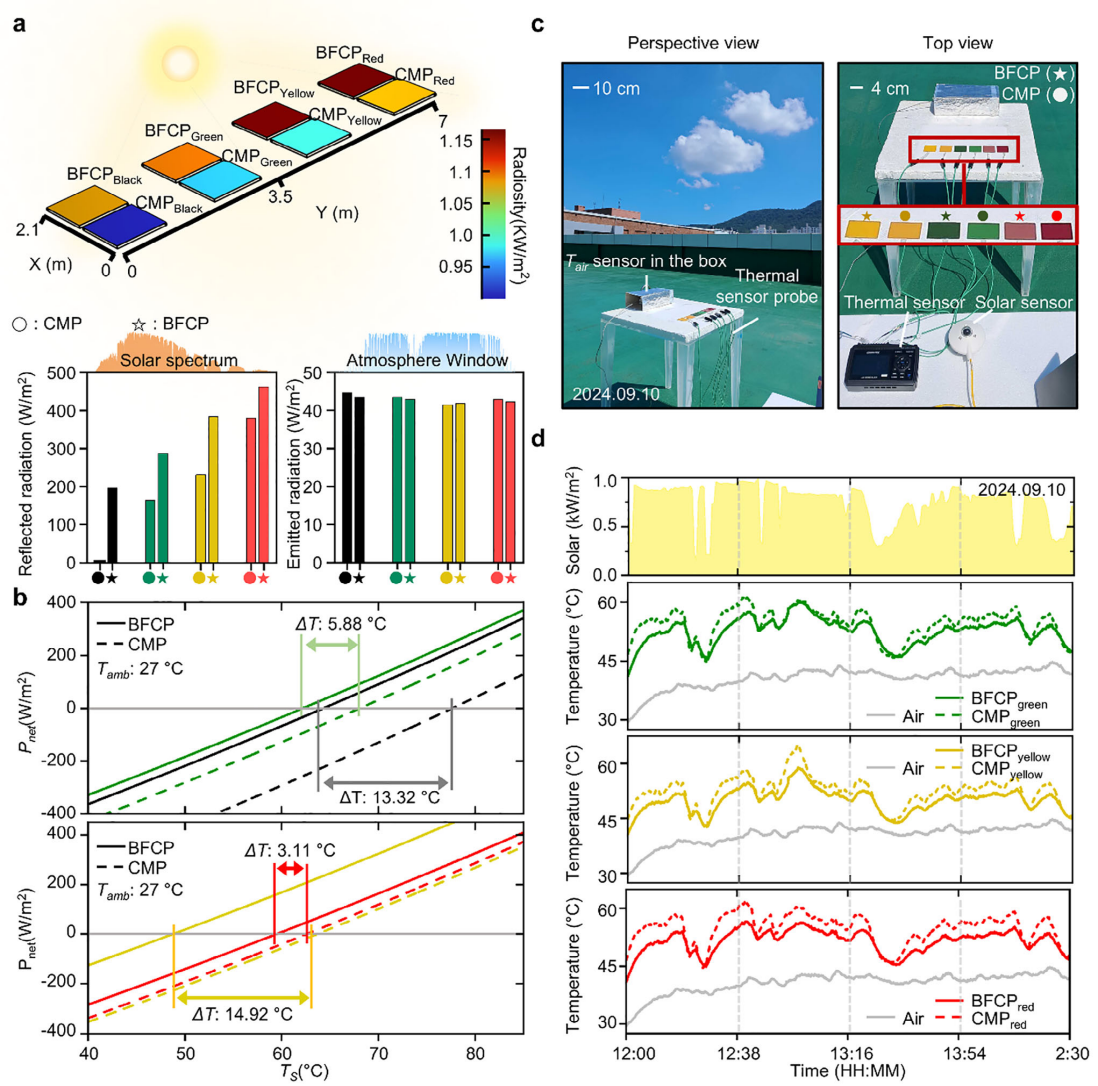
Radiative cooling (RC) effect of BFCP. a) Schematic of optical simulation of BFCP and CMP. Using optical simulation in COMSOL, the radiosity radiated from the film surface can be compared between BFCP and CMP. Additionally, the reflected radiation within the solar spectrum and the emitted radiation in LWIR range (e.g., 8–13 µm) are analyzed. b) Graph of the net cooling power. The thermal equilibrium equation, which accounts for the factors influencing the samples (i.e., BFCP and CMP), enables the estimation of its net cooling power and thermal equilibrium temperature. c) Configuration of the outdoor cooling test. d) Graph of the temperature of the sample in the outdoor cooling test. Under a specific solar irradiance (kW/m^2^), as a result of simultaneous temperature measurements of BFCP and CMP samples, BFCP exhibited a greater cooling effect than CMP.

The RC effect refers to the process of blocking solar energy at the surface within the solar spectrum and dissipating it through thermal emittance in the LWIR range.^[^ ^6^, ^7^, ^8^, ^9^, ^10^ ^]^ To evaluate these effects between BFCP and CMP, optical simulations were conducted. **Figure** **5**a illustrates the radiosity flux leaving a surface of a metal sheet per unit area, which is the sum of reflected, emitted, and transmitted fluxes on an oblique surface.^[^ ^33^, ^34^, ^35^ ^]^ Since the transmitted flux is negligible, the radiant flux to the exterior can be estimated using radiosity. As shown in Figure 5a, BFCP with various colors exhibits enhanced radiosity compared to CMP with similar colors. This is attributed to the higher reflected radiation of the BFCP surface in the solar spectrum, while its emitted radiation in the LWIR range remains comparable to that of CMP.

In addition to analyzing radiosity, a thermal equilibrium equation analysis in the Method section was conducted to infer the surface temperature resulting from the RC effect.^[^ ^36^, ^37^, ^38^ ^]^ Using this equation, the thermal equilibrium temperature of the sample was determined by accounting for the solar energy absorbed by the sample, the heat radiation emitted from the sample with a specific temperature, and its interaction with the surrounding atmosphere. Figure 5b describes that regarding the thermal equilibrium temperature, BFCP generally exhibits a lower temperature than CMP. Among the colors, yellow, which has the greatest difference in radiosity, demonstrates the largest temperature difference (i.e., Δ*T* = 14.92 °*C*), whereas red, which exhibits the smallest difference, has the least temperature variation (i.e., Δ*T* = 3.11 °*C*).

In the outer experiment, we performed the temperature of the sample test. As shown in Figure 5c, under strong sunlight (Solar irradiance ≤ 1.0 kW m^−2^), the temperature of BFCP and CMP were measured using a temperature sensor for each color, while the air temperature (*T_air_*) was recorded using an air temperature sensor. Figure 5d illustrates that *T_air_* is higher than the ambient temperature due to the heating effect of the box enclosing the air sensor under the strong sunlight.^[^ ^38^ ^]^ Additionally, environmental factors such as wind and fine dust introduce slight deviations from simulation results. Nevertheless, when analyzed by color, BFCP exhibits a lower temperature than CMP (i.e.*, ΔT_green_* ≤ 6.5 °C, *ΔT_yellow_* ≤ 6.9 °C, and *ΔT_red_* ≤ 6.8 °C). This can be attributed to the superior NIR reflectance of BFCP in comparison to CMP. Furthermore, the temperature analysis with respect to saturation levels, as shown in Figure S8 (Supporting Information), indicates that the black–colored BFCP (i.e., wt.%: 100%) exhibited a lower temperature than CMP (i.e.*, ΔT_black_* ≤ 6.4 °C). Additionally, as the wt.% decreases, the temperature further decreases. This cooling effect is attributed to the higher weight fraction of white pigment in black–colored BFCP, which enhances NIR reflectance while simultaneously increasing overall visible reflectance, thereby contributing to a lower sample temperature.

Additionally, as described in the Methods section, BFCP, and CMP were utilized as color‐based coats on galvanized steel sheets instead of glass substrates, and a temperature experiment was conducted. As shown in Figure S9 (Supporting Information), consistent with the previous case, BFCP exhibited a superior cooling effect compared to CMP (i.e.*, ΔT*: 3.9–9.5 °C), thereby demonstrating its potential applicability in real automotive vehicles.

### 2.5 LiDAR Detection Test

**Figure 6:**
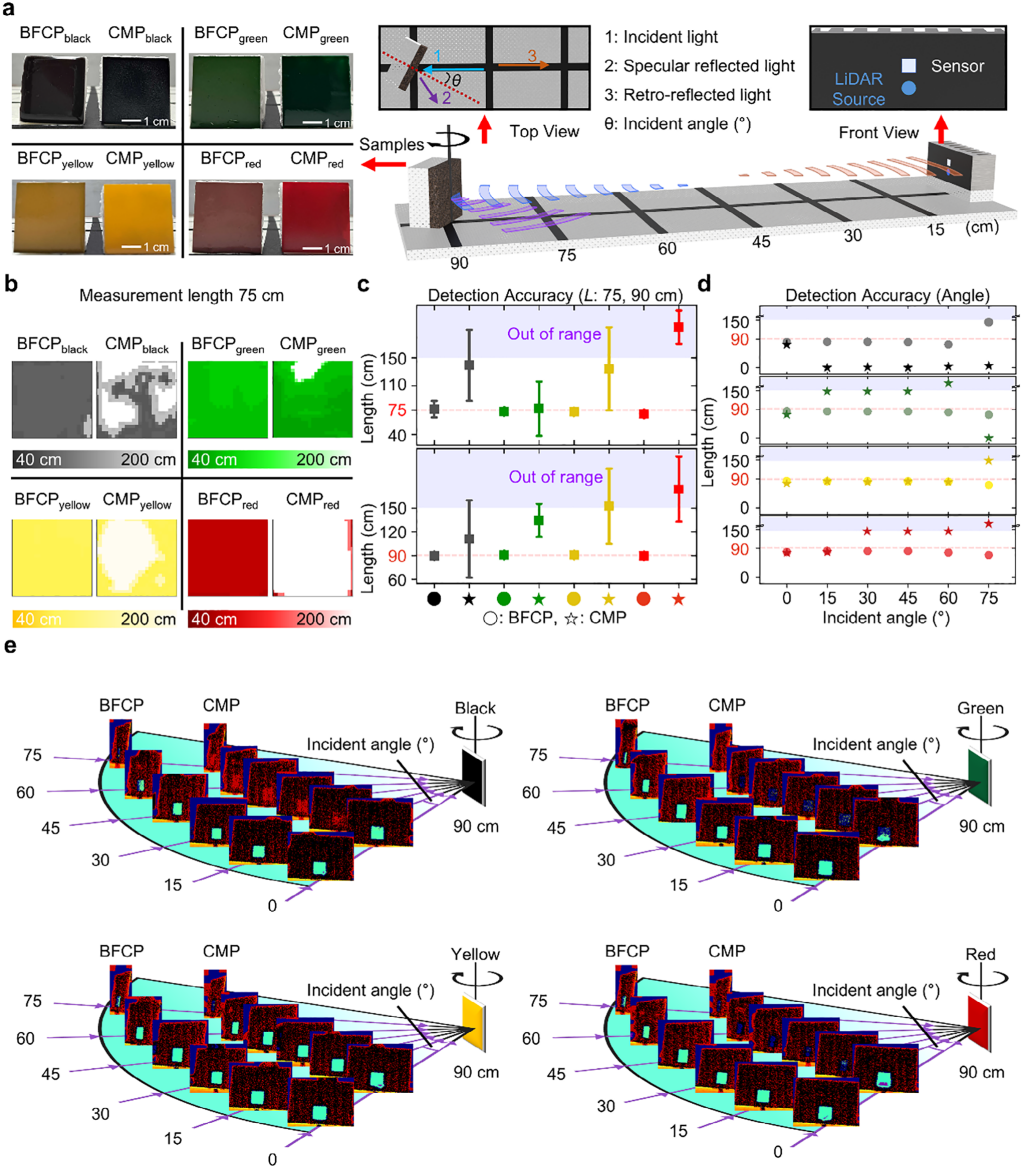
LiDAR detection of BFCP. a) Illustration of the LiDAR detection experiment setup. LiDAR emits 940 nm wavelength light toward an object and calculates the distance based on the time of flight (ToF) of the reflected signal. b) Mapping process from detected data at a measurement length of 75 cm. Figure S11 (Supporting Information) is a mapping image at a measurement length of 90 cm. c) Graph of detection accuracy depending on measurement length (*L*). In the LiDAR detection range (e.g., 0–150 cm), by calculating the average and standard deviation of the detected data, the difference in detection capability between BFCP and CMP can be evaluated. d) Graph of detection accuracy depending on the incident angle. Based on Figure 6e, by measuring the minimum detected distance, changes in the detection capability of BFCP and CMP were observed as a function of the incident angle. e) Illustration of LiDAR detection outcomes relative to the incident angle.

**Figure** **6**a presents a comparative experimental setup with CMP to evaluate the LiDAR detection capability of BFCP. The experimental setup positions the object at a specific distance (*L*) from the LiDAR system installed at one end. When a 940 nm wavelength light is emitted from the source toward the object, the returning light is detected by the sensor, this experiment aims to compare the detected areas to evaluate detection performance. Furthermore, the detected area was examined by rotating the object at a specific angle, allowing for a comparative analysis based on the incident angle.

First, as shown in Figure S10 (Supporting Information), the detected area was compared based on the distance (e.g.*, L* = 75 and 90 cm). Figure 6b presents the result of a new mapping process from detected data. Figure S11 (Supporting Information) corresponds to the case with a distance of 90 cm. Figure 6c illustrates that, under the LiDAR detection setting (i.e., 0–150 cm), the BFCP is detected at a distance close to the actual target position on average, with a relatively small standard deviation. In contrast, CMP is, on average, perceived as being farther from the actual target position. Additionally, the deviation is large, and some detected points exceed the detection range.

Second, Figure S12 (Supporting Information) illustrates that LiDAR emits light toward the object at various incident angles (°) by rotating the object on the angular sheet. Based on the detected area data obtained for various incident angles (Figure 6e), As illustrated in Figure 6d, an increase in the incident angle makes it challenging to detect specularly reflected light as it moves beyond the sensor range. However, BFCP effectively generates retro‐reflected light, resulting in a minimum detected distance value that closely matches the actual target distance. However, CMP does not effectively generate retro‐reflected light. As a result, for black and green colors, the minimum detected distance value significantly deviates from the actual target distance. In the case of yellow and red, when the incident angle exceeds a certain threshold, a discrepancy arises between the minimum detected distance value and the target distance. In extreme cases, detection fails entirely.

To more clearly evaluate the effectiveness of LiDAR detection of BFCP, three distinct experiments were conducted. As described in Figures S13–S17 (Supporting Information): 1) LiDAR measurements using an Ag mirror, 2) LiDAR measurements under a spherical coordinate setup, and 3) LiDAR measurements in a highly humid environment mimicking fog. The first experiment provided the key insight that enhancing backscattering‐based reflectance is more critical than simply increasing total reflectance arising from specular reflection. Furthermore, the results of the second and third experiments demonstrate that BFCP exhibits greater stability under diverse environmental conditions, describing reduced sensitivity to external factors compared to reference materials (e.g., CMP and Ag mirror).

## 3 Conclusion

This study investigates BFCP for the development of automotive pigment optimized for energy‐efficient future autonomous vehicles. CMPs primarily focus on aesthetic appeal and do not effectively mitigate in‐vehicle temperature rise on hot days, leading to inefficiencies in vehicle cooling energy consumption. Additionally, they have the drawback of inconsistent LiDAR recognition depending on environmental conditions. To address these issues, metal‐oxide ceramic materials were utilized to enhance thermal emissivity by leveraging resonance within ionic bonds in the LWIR range. Additionally, doping with Mn^4+^, Ti^4+^, and Ta^5+^ ions through the calcination process induces optical structural modifications. As a result of these altered optical properties, the absorption coefficient decreases (i.e.*, k* < 0.1), while the refractive index contrast with PDMS increases within the NIR range (i.e.*, Δn*  ≥  1.1). Consequently, the RC effect is achieved through enhanced reflectance in the NIR band due to increased scattering of incoming light. In addition, from a structural perspective, the pigment exhibits a particle structure with a comparably optimal size determined through Mie theory, effectively inducing back‐scattered waves. This ultimately enables LiDAR detection even at varying external factors. As a result, BFCP has the potential to serve as a next‐generation automotive pigment with dual functionality.

By expanding its applicability, as shown in Figure S18a (Supporting Information), the BFCP_black_ demonstrates lower NIR reflectance compared to other colors, which results in a distinct difference under the IR image. This property enables the vehicle surface to maintain a visually colorful appearance, while selectively revealing hidden patterns in the IR image, as demonstrated by BFCP_black_. Furthermore, in Figure S18a (Supporting Information), the difference between BFCP and CMP is clearly distinguishable in the IR image for colors other than black. This indicates that even when BFCP and CMP are used together, the contrast in NIR reflectance can be leveraged to achieve anti‐counterfeiting effects (Figure S18b, Supporting Information).

## 4 Experimental Section

### Raw Materials

Metal oxide micro–nano particles were purchased that CaCO_3_ (99%, KOJUNDO, Japan), MnO_2_ (99%, KOJUNDO, Japan), TiO_2_ (99.99%, KOJUNDO, Japan), Cr_2_O_3_ (99.9%, KOJUNDO, Japan), Ta_2_O_5_ (99.9%, KOJUNDO, Japan), Bi_2_O_3_ (99.99%, KOJUNDO, Japan), V_2_O_5_ (>99%, JUNSEI, Japan), Fe_2_O_3_ (>95%, SAMCHUN, Korea), and Al_2_O_3_ (99.99%, SUMIMOTO, Japan), Ag (ITASCO, Korea). Polydimethylsiloxane (PDMS) was purchased from DOW CORNING (DC‐184, United States). The curling agent was purchased from DOW CORNING (SILASTIC RTV‐4234‐T4, United States). The commercial spray paints were purchased from ILSIN (Korea). The commercial paint pigments were purchased from KCOLOR (Korea). The liquid hide glue was purchased from Sukinikawa‐eki (Japan). The Acrylic urethane resin was purchased from APEC (Korea). Paint protection film (PPF, 3 M, United States). Siloxane‐based water‐repellent coating agent (BullsOne, Korea). The galvanized steel sheet from ALL STEEL (United States).

### Fabrication of BFCP

To fabricate optical structures that enhance NIR reflectance and improve LiDAR detection, as shown in Figure S3 (Supporting Information), the black series was doped with Mn in CaCO_3_ (i.e., CMO), the green series was doped with Ti in Cr_2_O_3_ (i.e., TCO), the yellow series was doped with Ta in BiVO_4_ (i.e., TBV), and the red series was doped with Ti in Fe_2_O_3_ (i.e., FT). Color pigments were synthesized using a conventional solid‐state reaction method.^[^ ^39^ ^]^ Each precursor was stoichiometrically measured and mixed in a mortar for 30 min. For example, black was mixed with CaCO_3_ powder (34.94 g) and MnO_2_ powder (15.06 g), green was mixed with Cr_2_O_3_ powder (47.98 g) and TiO_2_ powder (2.02 g), yellow was mixed with Bi_2_O_3_ powder (33.29 g), Ta_2_O_5_ powder (6.31 g) and V_2_O_5_ powder (10.4 g), and red was mixed with Fe_2_O_3_ powder (28.31 g) and TiO_2_ powder (21.69 g). Subsequently, they were calcined in alumina crucibles in an electric furnace. For black, green, yellow, and red, the calcination temperatures and durations were 1200 °C for 6 h, 1000 °C for 1 h, 800 °C for 9 h (twice), and 700 °C for 3 h respectively, with a heating rate of 10 °C min^−1^ (Figure S3, Supporting Information).

To fabricate the film of BFCP, a solution was prepared by mixing PDMS and BFCP pigment at a specific volume ratio. The solution mixed with 1% curling agent was then coated onto a soda‐lime glass substrate (4 cm  ×  4 cm) using the drop‐cast method and subsequently heated on a hot plate (e.g., 120 °C) for 3 min to complete the fabrication process. In addition, to achieve various saturation levels (Figure 4a), BFCP was mixed with white metal oxide micro–nano particles (Al_2_O_3_) at a specific mass ratio (wt.%) (Figure 4a; Tables S1–S3, Supporting Information). However, as shown in Figure S19 (Supporting Information), automotive painting procedures typically involve multiple coating layers.^[^ ^40^ ^]^ To fabricate the composite coating following the automotive painting procedure, a PDMS‐based BFCP layer was deposited onto a glass substrate as the color base coat and annealed at a typical processing condition^[^ ^41^ ^]^ (e.g., 120 °C for 3 min). Subsequently, an acrylic urethane resin was applied as the clear coat and cured under standard conditions (e.g., 150 °C for 20 min). On top of this structure, additional surface protection layers (e.g., PPF and glass coating) were applied. The glass coating was applied using a siloxane‐based water‐repellent coating agent.

Additionally, to fabricate a sample analogous to actual automotive paint (Figure S9a, Supporting Information), 4 g of acrylic urethane resin—which was used in automotive paint formulations—was employed instead of PDMS and mixed with BFCP. After thorough mixing and vacuum degassing, the resulting solution was uniformly deposited onto a galvanized steel sheet via a drop‐casting method. The coated sample was then annealed in an oven at 150 °C for 20 min, following a procedure consistent with standard automotive coating processes.^[^ ^41^ ^]^

### Structural Analysis of BFCP

To analyze of BFCP structure, the BFCP sample was observed under magnification, while its composition was analyzed using Scanning Electron Microscope/Energy Dispersive Spectroscopy (SEM/EDS, GEMINI500, ZEISS, Germany), and X‐ray Diffraction (XRD, XPERT 3, PANalytical, Netherlands) analysis confirmed the successful doping of BFCP.

### Optical Simulation and Calculation TOOL

The scattering efficiency by BFCP was simulated with commercial software, FullWAVE (Rsoft Design Group, Synopsys, United States) using the finite difference time domain (FDTD). When a plane wave was launched onto the spherical BFCP model, scattering was analyzed by examining the electronic field (E_y_), magnetic field (H_y_), and Poynting vector of the resulting scattered wave (Figure 2b,d). To investigate the correlation between size, volume ratio, and optical properties, simulations were conducted using the “MOST” function in Rsoft to calculate the Poynting vector of scattered waves outside the launch field. The simulations involved varying the circle diameter (i.e., 0.6–1.0 µm) and plane wave wavelength (i.e., 0.75–1.04 µm). Furthermore, to visualize the electromagnetic field distribution of scattered waves, simulations were conducted to compare the optimal BFCP circle diameter with other diameters (i.e., 0.3 and 3.0 µm), and to analyze multiple particles of optimal BFCP sizes. The results are presented in Figures S4 and S5 (Supporting Information).

To estimate the comparative analysis of the optical properties of BFCP and CMP, the heat transfer mode (COMSOL Multiphysics, United States) was used. In heat transfer mode, BFCP and CMP films (e.g., each 1 m  ×  1 m and 0.05 cm thick) were installed on an automotive steel plate (e.g., 1 m  ×  1 m of square and 5 cm thick). Under far‐field conditions, these models were then exposed to 1000 W of solar radiation in the ‐z direction. The results are presented in Figure 5a.

### Optical Characterization of BFCP

The BFCP was mixed with PDMS in specific proportions (Tables S1–S3, Supporting Information) and cured on a hot plate. The resulting film was used to measure the optical characterization of BFCP. The UV‐vis‐NIR spectrum (e.g., 200–2500 nm) was measured using a spectrophotometer (V770, JASCO, Japan), and the IR spectrum (e.g., 2.5–16.6 µm) using an FT‐IR spectrometer equipped with an integrating sphere (Nicolet 6700, Thermo‐Fisher Scientific, USA).

### Outdoor Temperature Test

As shown in Figure 5c, to conduct the outer temperature test, the solar sensor (CMP3, Kipp & Zonen B.V., Netherlands) was used to measure the solar irradiance, the thermal sensor (GL240, GRAPHTEC, Japan) to measure the temperature of samples, and air temperature sensor (GL240, GRAPHTEC, Japan) to measure the air temperature. Then, the samples were put on the acyl table with Styrofoam as an insulator to block the geothermal from the ground.

### LiDAR Test

As illustrated in Figure 6a, the LiDAR test setup was made as Styrofoam (e.g., 105 cm  ×  25 cm). On the Styrofoam, the LiDAR (CS20, DFROBOT, China) was attached at one end. And the film of BFCP or CMP which was attached to a square of Styrofoam (e.g., 4 cm  ×  4 cm), was fixed at a point in order of a certain distance. To make an incident angle (°), the film was rotated by a steel pedestal and rotation (SGSP‐60 YAW, SIGMAKOKI, Japan).

### Calculating Color Coordinates from Chromaticity Diagram

The color space was developed to model human color perception, incorporating chromatic aberration and light sensitivity. For this color space, colors are represented based on tristimulus values.

(1) $$x\;=100\frac{\int I\left(\lambda \right)R\left(\lambda \right)\bar{x}\left(\lambda \right)d\lambda }{\int I\left(\lambda \right)\bar{y}\left(\lambda \right)d\lambda }$$

(2) $$y\;=100\frac{\int I\left(\lambda \right)R\left(\lambda \right)\bar{y}\left(\lambda \right)d\lambda }{\int I\left(\lambda \right)\bar{y}\left(\lambda \right)d\lambda }$$

(3) $$z\;=100\frac{\int I\left(\lambda \right)R\left(\lambda \right)\bar{z}\left(\lambda \right)d\lambda }{\int I\left(\lambda \right)\bar{y}\left(\lambda \right)d\lambda }$$

where x, y, and z are the tristimulus values, $\bar{x}\left(\lambda \right)$, $\bar{y}\left(\lambda \right)$, and $\bar{z}\left(\lambda \right)$ are CIE color‐matching functions, *I*(λ) is CIE Illuminant D65 spectrum, *R*(λ) is the spectral reflectance of samples. Using the tristimulus values (x, y, and z), coordinates in the CIE 1931 color space of chromaticity are derived.

(4) $$X=\frac{x}{x+y+z}$$

(5) $$Y=\frac{y}{x+y+z}$$

where X and Y are coordinate values.

### Definition of Reflectance, and Thermal Emittance of Sample within a Specific Region

The reflectance $\left(\bar{R}\right)$ is defined as a ratio of the reflected solar intensity to the total incident solar intensity within a specific range (λ_1_ −λ_2_).

(6) $$\bar{R}=\frac{\int_{\lambda_{1}}^{\lambda_{2}}I_{AM1.5}\left(\lambda \right)R\left(\lambda \right)d\lambda }{\int_{\lambda_{1}}^{\lambda_{2}}I_{AM1.5}\left(\lambda \right)d\lambda }$$

where *I*_*AM*1.5_ is the AM 1.5 solar spectrum, *R*(λ) is the spectral reflectance of the sample.

Using Equation (6), the reflectance of a specific region is calculated. The thermal emittance $\left(\bar{\varepsilon }\right)$ is defined as a ratio of the spectral intensity to that of a standard blackbody at the same temperature within an LWIR region (8 − 13 µ*m*)

(7) $$\bar{\varepsilon }=\frac{\int_{8\mu m}^{13\mu m}I_{BB}\left(\lambda ,t\right)\varepsilon \left(\lambda ,t\right)d\lambda }{\int_{8\mu m}^{13\mu m}I_{BB}\left(\lambda ,t\right)d\lambda }$$

where *I_BB_* (λ,*T*) is the blackbody radiation, which is dependent on the temperature of the sample, ε(λ) is the spectral emittance of the sample.

### Radiosity

Radiosity (*J_e_*) is the radiant flux leaving a surface per unit area.

(8) $$J_{e}=\frac{\partial \Phi_{e}}{\partial A}=J_{e,em}+J_{e,r}+J_{e,tr}$$

where Φ_*e*_ is the radiant flux, *A* is the surface area, *J*_*e*,*em*_, *J*_*e*,*r*_, and *J*_*e*,*tr*_ are the emitted, reflected, and transmitted components of the radiosity. In the opaque surface, the transmitted component is negligible.

(9) $$J_{e}=J_{e,em}+J_{e,r}=\varepsilon \sigma T^{4}+\left(1-\varepsilon \right)E_{e}$$

where ε is the emissivity of the surface, σ is the Stefan–Boltzmann constant, *T* is the temperature of the surface, and *E_e_* is the irradiance of incident light.

The spectra radiosity (W/m^3^) was the radiosity of the surface per unit wavelength, which was the radiosity component in a specific wavelength.

(10) $$J_{e,\lambda }=\frac{\partial J_{e}}{\partial \lambda }$$

where *J*_*e*,λ_ is the spectra radiosity, λ is a wavelength (µm). Within a range (λ_1_–λ_2_), the radiosity (W/m^2^) could be calculated.

(11) $$J_{e}=\int_{\lambda 1}^{\lambda 2}\frac{\partial J_{e}}{\partial \lambda }d\lambda$$

### Radiative Cooling Power by Thermal Equilibrium

An object generally radiates energy as a blackbody (*I_BB_*).

(12) $$I_{BB}\left(\lambda ,T\right)=\frac{2hc^{2}}{\lambda^{5}}\frac{1}{e^{hc/k\lambda T}-1}$$

where *h* is the Planck constant, *c* is the speed of light in vacuum, and *k* is the Boltzmann constant.

Equation (12) describes that the peak of blackbody radiation exhibits a blue shift as the sample's temperature increases. Furthermore, the atmosphere also emits the light (ε_*atm*_).

(13) $$\varepsilon_{atm}\left(\lambda ,\theta \right)=1-t{\left(\lambda \right)}^{1/cos\theta }$$

where *t*(λ) is transmittance at the wavelength and in the zenith direction (θ).

The net cooling power (*P_net_*) is determined based on thermal equilibrium, which is derived from incident solar irradiance, the sample's radiance, and its interaction with an atmosphere or surrounding environment.

(14) $$P_{net}\left(T_{S}\right)=P_{rad}\left(T_{S}\right)-P_{atm}\left(T_{amb}\right)-P_{sun}-P_{non-rad}$$

where *T_S_* is sample's temperature, *T_amb_* is ambient temperature, *P_rad_* is sample radiation power, *P_atm_* is radiation power from the atmosphere, *P_sun_* is incident solar power, *P*_*non* − *rad*_ is non‐radiation power from the surrounding environment such as conduction or convection.

Equation (14) determines the thermal equilibrium temperature at which the net cooling power is zero. Each power component is derived from the following expressions.

(15) $$P_{rad}\left(T_{S}\right)=\int \int_{0}^{\infty }\left[\varepsilon_{s}\left(\lambda ,\theta \right)I_{BB}\left(\lambda ,T_{S}\right)\right]d\lambda \cos\theta d\Omega$$

(16) $$P_{atm}\left(T_{amb}\right)=\int \int_{0}^{\infty }\left[\varepsilon_{s}\left(\lambda ,\theta \right)\varepsilon_{atm}\left(\lambda ,\theta \right)I_{BB}\left(\lambda ,T_{amb}\right)\right]d\lambda \cos\theta d\Omega$$

(17) $$P_{sun}=\int_{0}^{\infty }\left[\varepsilon_{s}\left(\lambda ,\theta \right)I_{AM1.5G}\left(\lambda \right)\right]d\lambda$$

(18) $$P_{non-rad}=h_{non-rad}\left(T_{amb}-T_{s}\right)$$

where ε_*s*_(λ,θ) is the spectral emittance of the sample in the zenith direction (θ), Ω is steradian, *I*_*AM*1.5*G*_(λ) is the AM 1.5 solar spectrum, and *h_non‐rad_* is the non‐radiative heat transfer coefficient (8 W m^−2^k).

Equation (15) demonstrates that the sample radiates at a specific temperature as a blackbody in a diverse direction. Moreover, Equation (16) an atmosphere radiates to the sample, which was in the LWIR range (8–13 µm) the atmosphere effect on the sample was minimal due to the high transmittance of the atmosphere. According to Equation (17), the incident solar power on the sample was calculated within a solar spectrum. Non‐radiation power (Equation 18), including conduction and convection, was generally estimated based on standard assumptions (i.e.*, h_non‐rad_*).

## Conflict of Interest

The authors declare no conflict of interest

## Author Contributions

I.H. presented methodology the that conducted the experiment and collected data. Y.‐J., J.S., and G.H.  fabricated the pigment and conducted optical property measurements. Y.J. proposed production methods and provided guidance on illustration. G.J. supervised the study, reviewed the manuscript, and contributed to its revisions.

## Supporting information



Supporting Information

## Data Availability

The data that support the findings of this study are available in the supplementary material of this article.
