# Supplementary material for: Dual‐Function Ceramic Pigments for Energy‐Efficient and Secure Autonomous Vehicles
Source: Adv Sci (Weinh). 2025 Apr 30;12(27):2503901. doi: 10.1002/advs.202503901 (PMC12279191; doi:10.1002/advs.202503901)
Supplement: Supplementary file 1 — Supporting Information [file ADVS-12-2503901-s001.docx]

Supplementary information

**Dual-Function Ceramic Pigments for Energy-Efficient and Secure Autonomous Vehicles**

Ik Hoon Jeong^1†^, Yong-jun Seo^2†^, Jum Soo Hwang^3^, Geun hyeong Kim^3^, Yeong Jae Kim^2,*^, and Gil Ju Lee^1,*^

†: These authors are contributed equally this work

*Corresponding authors: Gil Ju Lee ([gjlee0414@pusan.ac.kr](mailto:gjlee0414@pusan.ac.kr)), Yeong Jae Kim ([kimyj@kicet.re.kr](mailto:kimyj@kicet.re.kr))

**This Supplementary includes:**

**Methods S1 to S4**

**Figures. S1 to S20**

**Tables S1 to S4**

**Reference**

**Methods**

**Method S1. Ceramic-based doping approach for optical property enhancement.**

Ceramic material leads to high thermal emission with visible coloration, making such materials well-suited for our intended applications. To achieve distinct coloration while enhancing NIR reflectance, in accordance with the objectives of the study, ceramic-based doping was applied to the material.

To fabricate a black color with high NIR reflectance, we first doped CaCO_3_ with MnO_2_, which imparts a dark appearance due to the presence of Mn^4+^ ions. However, the electrical properties of the material—such as bandgap and ion transitions—are known to vary depending on the doping concentration of Mn^4+^, which in turn affects the NIR reflectance^1^.

For a green color with high NIR reflectance, we doped Cr₂O₃—which naturally exhibits a green hue—with TiO₂. The Ti^4+^ ions are expected to interact with the free electrons in Cr_2_O_3_, which reduces the electrical conductivity of material (*i.e.,* $\sigma$). This reduction in conductivity suppresses free-carrier absorption, thereby enhancing the NIR reflectance. However, similar to the Mn^4+^ case, the introduction of Ti^4+^ ions alters the bandgap characteristics of the material. Therefore, we systematically adjusted the Ti^4+^ doping concentration to optimize the NIR performance^2^.

Furthermore, a yellow-colored BiVO_4_ compound was synthesized through a mixing process of Bi_2_O_3_ and V_2_O_5_. To improve reflectance, Ta_2_O_5_ was doped into the compound. The incorporation of Ta^5+^ ions induces lattice distortion within the VO_4_ tetrahedra, which suppresses electron-hole pair recombination. This suppression leads to an increase in the dielectric constant (*i.e.,* $\varepsilon$) and subsequently enhances the NIR reflectance. In addition, higher concentrations of Ta^5+^ doping were found to reduce the particle size, which was utilized to tune structural properties for enhanced Mie scattering. Therefore, we performed an optimization of the Ta^5+^ doping concentration to improve optical performance^3^.

Finally, to achieve a red-colored material with high NIR reflectance, we doped Fe_2_O_3_ which reflects red wavelengths in the visible region with TiO_2_, known for high NIR reflectance. However, the incorporation of Ti^4+^ ions modifies the bandgap characteristics which affects reflectance in the visible range. Therefore, the Ti⁴⁺ doping concentration was carefully adjusted to maintain the red appearance while improving NIR performance^4^.

In conclusion, to enhance the NIR reflectance for each color-specific ceramic material, we systematically varied the doping weight ratios (*i.e.,* *wt%*) as described in Table S4. By varying the doping concentrations of Mn^4+^, Ti⁴⁺ and Ta⁵⁺ ions, we successfully fabricated materials exhibiting optimized optical performance, as illustrated in Figure S20.

**Method S2. Mie theory based on Maxwell’ equation.**

Mie theory provides a set of equations to effectively analyze the interaction between an EM wave ($\lambda$) and a spherical particle ($r$). The interaction is governed by two independent non-dimensional parameters^5-7^, such as 1) complex refractive index ($m=n-ik$) and 2) particle size parameter ($\chi=2\pi r/\lambda$). Specifically, when a particle is embedded in a material with a refractive index ($n_{0}$), the effective complex refractive index is expressed as $m^{'}=(n-ik)/n_{0}$. Based on two parameters, the relationship between the incident wave and the scattered radiation can be described using the complex amplitude functions ($S_{1}$, $S_{2}$)^5^.

| $S_{1}(\theta)=\sum_{n=1}^{\infty} \frac{2n+1}{n(n+1)}\left[ a_{n}\pi_{n}\left( \theta\right)+b_{n}\tau_{n}(\theta) \right]$ | (1) |
| --- | --- |
| $S_{2}(\theta)=\sum_{n=1}^{\infty} \frac{2n+1}{n(n+1)}\left[ a_{n}\tau_{n}\left( \theta\right)+b_{n}\pi_{n}(\theta) \right]$ | (2) |

where $\theta$ represents the angle between the direction of the incident and scattered waves, $n$ represents the order of the Bessel function, $a_{n}$,$b_{n}$ are the complex scattering Mie coefficients, and $\pi_{n}\left( \theta\right), \tau_{n}\left( \theta\right)$ are the angular functions. Using Equations (1) and (2), the direction and intensity of scattered radiation are estimated.

This relationship is derived by converting Maxwell’s equations into a spherical coordinate ($r, \theta, \phi$) From the results, the radial components ($a_{n}$,$b_{n}$) and angular characteristics ($\pi_{n}\left( \theta\right), \tau_{n}\left( \theta\right)$) of scattering can be determined using Bessel functions.

| $a_{n}=\frac{\psi_{n}^{'}\left( m\chi\right)\psi_{n}\left( \chi\right)-m\psi_{n}(m\chi)\psi_{n}^{'}(\chi)}{\psi_{n}^{'}\left( m\chi\right)\zeta_{n}\left( \chi\right)-m\psi_{n}(m\chi)\zeta_{n}^{'}(\chi)}$ | (3) |
| --- | --- |
| $b_{n}=\frac{{m\psi}_{n}^{'}\left( m\chi\right)\psi_{n}\left( \chi\right)-\psi_{n}(m\chi)\psi_{n}^{'}(\chi)}{{m\psi}_{n}^{'}\left( m\chi\right)\zeta_{n}\left( \chi\right)-\psi_{n}(m\chi)\zeta_{n}^{'}(\chi)}$ | (4) |

where $\psi_{n}, \zeta_{n}$ are the Riccati-Bessel functions which define spherical Bessel functions, $\psi_{n}^{'}$ represents the first derivation of $\psi_{n}$.

Also, the angular functions ($\pi_{n}\left( \theta\right), \tau_{n}\left( \theta\right))$ are determined through recurrence relations.

| $\pi_{n}\left( \theta\right)=\frac{2n-1}{n-1}\cos\theta\pi_{n-1}(\theta)-\frac{n}{n-1}\pi_{n-2}(\theta)$ | (5) |
| --- | --- |
| $\tau_{n}\left( \theta\right)=n\cos\theta\pi_{n}(\theta)-(n+1)\pi_{n-1}(\theta)$ | (6) |
| $\pi_{0}\left( \theta\right)=0, \pi_{1}\left( \theta\right)=1$ | (7) |

The above calculation codes utilized were the same as those employed in the previous study^8,10^.

Accordingly, scattering by specific particles can be predicted using Maxwell’s equation.

**Method S3. Scattered radiation from nano- and micro-sized particles.**

When a propagating electromagnetic wave (EM wave) interacts with a spherical particle, the EM wave is transmitted through the particles; however, a portion of their intensity is attenuated during the interaction. The attenuated intensity is quantified as the extinction coefficient of the particle^5^. This intensity can be represented as the extinction cross-section ($C_{ext}$) in a spherical coordinate system ($r, \theta, \phi$). The extinction cross-section is divided into two components, as the attenuated intensity of a particle is attributed to scattering and absorption.

| $C_{ext}=C_{sca}+C_{abs}$ | (8) |
| --- | --- |

where $C_{sca}$, $C_{abs}$ are cross-sections of scattering and absorption.

The effectiveness of a particle’s interaction with an EM wave can be evaluated through its efficiency relative to the intensity of the incident wave.

| $Q_{ext}=\frac{C_{ext}}{\pi r^{2}}=\frac{2}{\chi^{2}}\sum_{n=1}^{\infty} \left( 2n+1 \right)Re\{a_{n}+b_{n}\}$ | (9) |
| --- | --- |
| $Q_{sca}=\frac{C_{sca}}{\pi r^{2}}=\frac{2}{\chi^{2}}\sum_{n=1}^{\infty} \left( 2n+1 \right)Re(\left\vert a_{n} \right\vert^{2}+\left\vert b_{n} \right\vert^{2})$ | (10) |

where $Q_{sca}$ represents scattering efficiency, $\pi r^{2}$ the cross-section of a particle.

As shown in Equation (9) and (10), the efficiency is calculated as the ratio of the cross-section to the incident wave interacting with the particle. Each cross-section can be determined using the scattering coefficient ($a_{n}, b_{n}$), while the absorption efficiency can be calculated by combining Equation (8) and (9). Otherwise, the directionality of the scattered wave can be determined using the complex amplitude functions ($S_{1}$, $S_{2}$) in Mie theory based on Maxwell’ equation part.

| $\Phi(\theta)=2\frac{i_{1}+i_{2}}{\chi^{2}Q_{sca}}$ | (11) |
| --- | --- |
| $i_{1}={\vert S_{1}(r,\theta,\phi)\vert}^{2}, i_{2}={\vert S_{2}(r,\theta,\phi)\vert}^{2}$ | (12) |

where $\Phi(\theta)$ represents the phase function, $i_{1,2}$ represents the polarized intensities.

The directionality of the scattered wave from a particle is estimated using the phase function, which represents the ratio between the total scattering intensity and the intensity of scattering in a specific direction (Equation (11) and (12)). Consequently, scattering efficiency ($Q_{sca}$) provides essential information about the particle with the highest scattering performance. Additionally, the phase function ($\Phi(\theta)$) facilitates the prediction of scattering direction as a function of particle size (r). The above calculations in the Mie theory have been implemented in the previous code^10^.

In the heterogeneous substrate containing randomly distributed particles, the effective scattering coefficient ($\sigma_{s}$) and effective phase function ($\Phi_{T}(\theta))$ are estimated based on scattering cross-section, under the assumptions of independent scattering and uniform dispersion of particles^5,6^.

| $\sigma_{s}=\sum_{i=1}^{m} \pi r_{i}^{2}N_{i}Q_{sca, i}$ | (13) |
| --- | --- |
| $\Phi_{T}(\theta)=\frac{1}{\sigma_{s}}\sum_{i=1}^{m} \pi r_{i}^{2}N_{i}Q_{sca, i}\Phi(r_{i},\theta)$ | (14) |

where $i$ is the order of particles ($i:1,2,3\ldots m$), $\pi r_{i}^{2}Q_{sca, i}$ represents the cross-section of a particle having $i_{th}$ radius ($r_{i})$, $N_{i}$ is the number of particles having $i_{th}$ radius ($r_{i})$ per unit volume.

Based on the Equation (13), (14), we can approximate the effective scattering coefficient and phase function.

| $f_{i}=\frac{4}{3}\pi r_{i}^{3}N_{i}$ | (15) |
| --- | --- |
| $\sigma_{s}=\sum_{i=1}^{m} \frac{3f_{i}}{4r_{i}}Q_{sca, i}$ | (16) |
| $\Phi_{T}(\theta)=\frac{1}{\sigma_{s}}\sum_{i=1}^{m} \frac{3f_{i}Q_{sca, i}}{4r_{i}}\Phi(r_{i},\theta)$ | (17) |

where $f_{i}$ is volume concentration of particles

By knowing the volume concentration ($f_{i}$) of particles for each radius ($r_{i}$), the scattering properties of heterogenous substrates can be estimated.

**Method S4. Backscattering.**

As illustrated in Mie theory based on Maxwell’ equation in the Method S2, using the complex amplitude functions ($S_{1}$, $S_{2}$), the direction and intensity of the scattered wave can be estimated. These functions were also employed to calculate the phase function as described in the scattered radiation from nano- and micro- sized particles in the Method S3. For the scattered wave directed toward retro-reflection, the polarized intensity at 180 °is related to scattering amplitude vector *S*(180 °), expressed as $i\left( \chi,m^{'},\theta\right)={|S|}^{2}$. In the case of a hypothetical isotropic scatterer, the backscattering cross-section, which corresponds to intensity in a spherical coordinate system ($r, \theta, \phi$), is determined by this vector^9^.

| $C_{b}=4\pi\frac{{\vert S({180}^{\circ})\vert}^{2}}{\kappa^{2}}$ | (18) |
| --- | --- |

where $C_{b}$ represents the backscattering cross-section, $\kappa= \frac{2\pi}{\lambda}$ is wavenumber.

$S({180}^{\circ})$ can be obtained through vessel function calculations for the polarization of light in Mie theory based on Maxwell’ equation part of the Method S2.

| ${\vert S({180}^{\circ})\vert}^{2}={\vert S_{1}({180}^{\circ})\vert}^{2}\cos^{2} \phi+{\vert S_{2}({180}^{\circ})\vert}^{2}{sin}^{2} \phi$ | (19) |
| --- | --- |
| $S_{2}\left( {180}^{\circ} \right)=-S_{1}\left( {180}^{\circ} \right)=\frac{1}{2}\sum(2n+1)\left( -1 \right)^{n}(a_{n}-b_{n})$ | (20) |

where $\phi$ is phase of EM wave.

Accordingly, by combining Equation 18, 19, and 20, the backscattering cross-section can be derived. Furthermore, the backscattering efficiency ($Q_{b}$) can be determined as a function of particle size such as Equation (9), (10).

| $C_{b}=\frac{\pi}{\kappa^{2}}\left\vert\sum\left( 2n+1 \right)\left( -1 \right)^{n}\left( a_{n}-b_{n} \right) \right\vert^{2}$ | (21) |
| --- | --- |
| $Q_{b}=\frac{C_{b}}{\pi r^{2}}=\frac{1}{\chi^{2}}\left\vert\sum\left( 2n+1 \right)\left( -1 \right)^{n}\left( a_{n}-b_{n} \right) \right\vert^{2}$ | (22) |

The above calculations in the Mie theory have been implemented in the previous code^10^.

In systems with multiple particles, As shown in the scattered radiation from nano- and micro-sized particles part of Method S3, the backscattering from a heterogeneous substrate can be estimated by analyzing the backscattering direction in the effective phase function (Equation 17).

| $\Phi_{T}(\theta)=\frac{1}{\sigma_{T}}\sum_{i=1}^{m} \frac{3f_{i}Q_{sca,i}}{4r_{i}}2\frac{{\vert S_{1}\vert}^{2}+{\vert S_{2}\vert}^{2}}{{\chi_{i}}^{2}Q_{sca,i}}$ | (23) |
| --- | --- |
| $\Phi_{T}({180}^{^{\circ}})=\frac{1}{\sigma_{T}}\sum_{i=1}^{m} \frac{3f_{i}}{4r_{i}}\frac{1}{{\chi_{i}}^{2}}\left\vert\sum\left( 2n+1 \right)\left( -1 \right)^{n}\left( a_{n}-b_{n} \right) \right\vert^{2}$ | (24) |
| $\Phi_{T}\left( {180}^{^{\circ}} \right)=\frac{1}{\sigma_{T}}\sum_{i=1}^{m} \frac{3f_{i}Q_{b,i}}{4r_{i}}$ | (25) |

From Equation 25, the intensity of backscattering in a heterogeneous substrate can be calculated based on the volume concentration ($f_{i}$) and backscattering efficiency ($Q_{b,i}$) of particles with varying sizes ($r_{i})$ (*i.e.,* $\sum_{i=1}^{m} \frac{3f_{i}Q_{b,i}}{4r_{i}})$. Moreover, in relation to the effective scattering coefficient ($\sigma_{T}$), the proportion of backscattering ($\Phi_{T}\left( {180}^{^{\circ}} \right)$) within the substrate can be estimated.

**Figures**

**
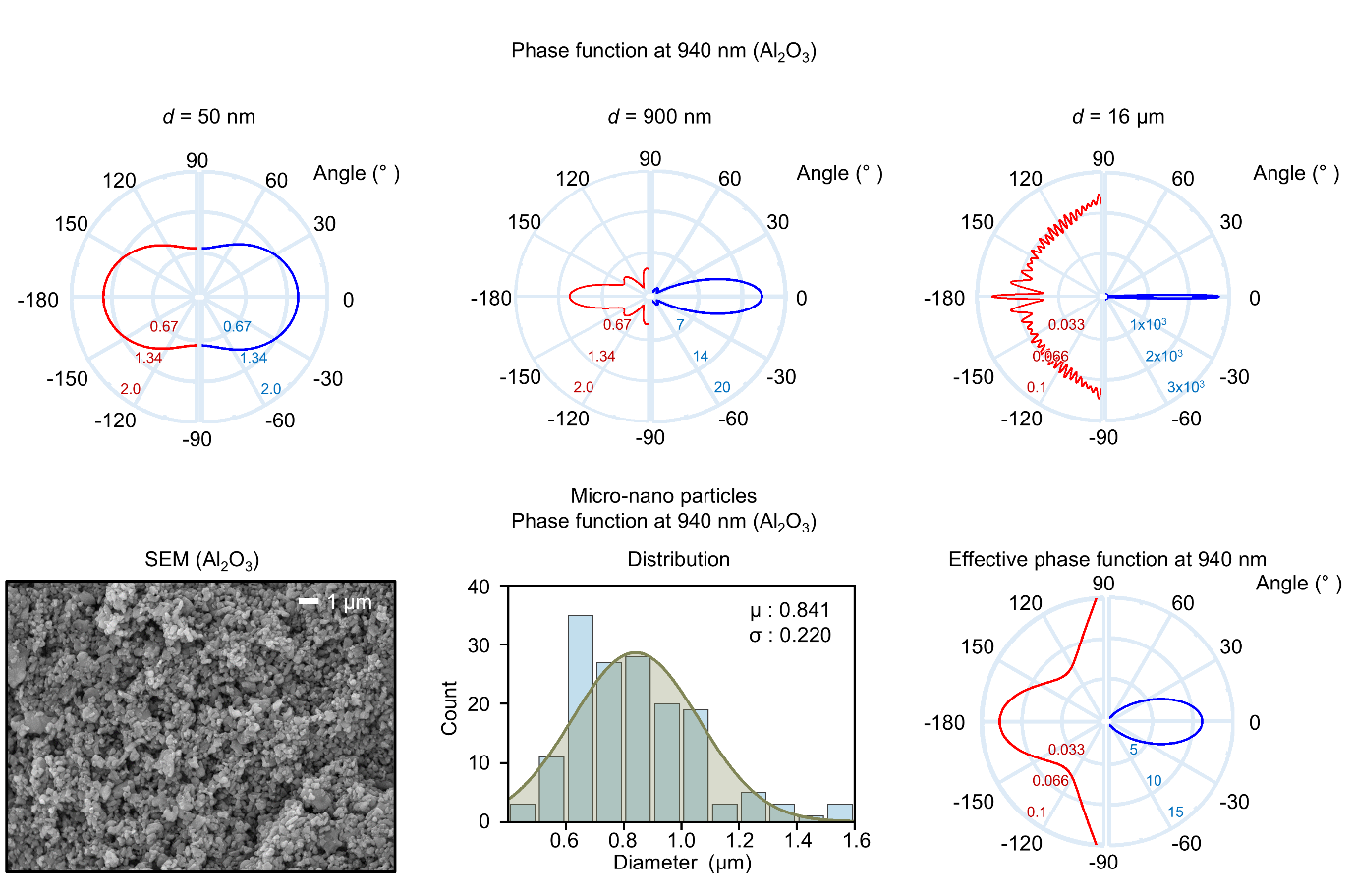
**

**Figure S1.** Phase function of Al_2_O_3_ micro–nano particles. Based on the optical characteristic of Al_2_O_3_, it is observed that the types of scattering vary depending on the particle size. First, for particle sizes (*d)* much smaller than the incident wavelength (*wl*) as described by Rayleigh (*e.g.,* *d* = 50 nm and *wl* = 940 nm), the scattered wave exhibits nearly equal forward and backward propagation. And for particle sizes (*d)* much larger than the incident wavelength (*wl*) as described by Geometric (*e.g.,* *d* = 16 μm and *wl* = 940 nm), the scattered wave is primarily directed forward. However, for particle sizes (*d)* comparable to the incident wavelength (*wl*) as described by Mie (*e.g.,* *d* = 900 nm and *wl* = 940 nm), the overall scattering intensity exceeds that of Rayleigh scattering. As in Geometric scattering, forward scattering is dominant compared to Mie scattering; however, Mie scattering exhibits pronounced backscattering (*i.e.,* *−*180 °) in the backward direction, leading to directional propagation rather than diffuse dispersion. For example, scanning electron microscopy (SEM) analysis of Al_2_O_3_ powder suggests that the powder consists of Mie**–**scale particles, leading to enhanced effective backscattering.

**
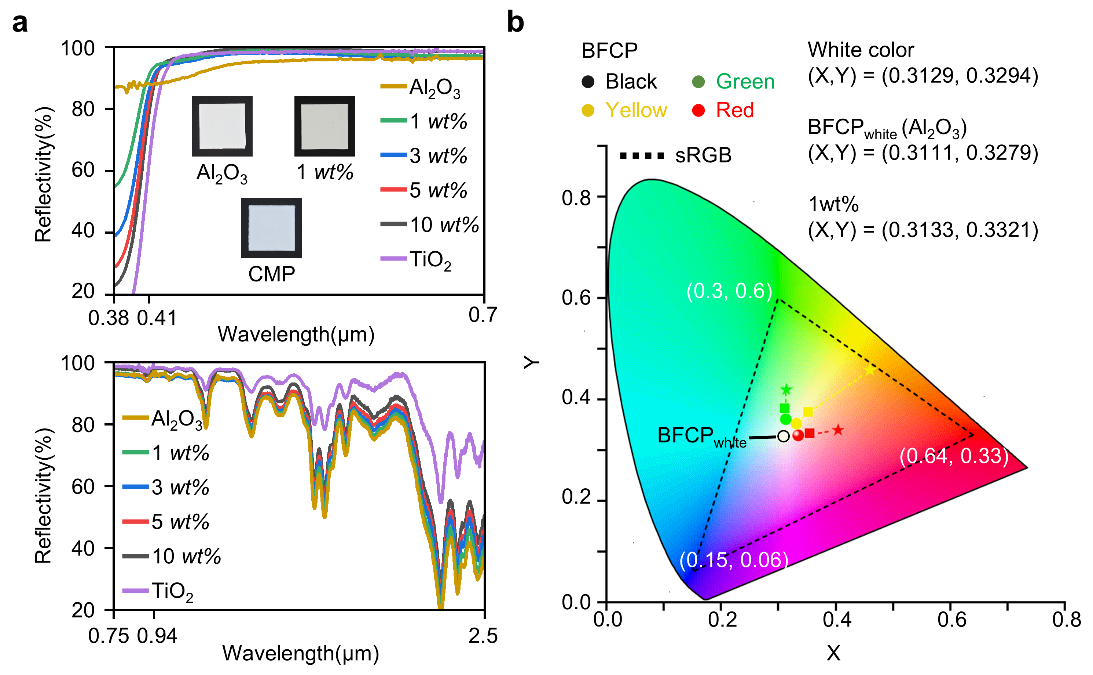
**

**Figure S2.** Spectral and optical properties of white-color pigments. a) Graph of the optical characteristics of white-color ceramic pigments. While Al_2_O_3_ inherently reflects more than 85 % of light in the visible spectrum, making it appear white. However, its reflectance significantly declines in the NIR region at longer wavelengths. To overcome this drawback and achieve reflectance exceeding 90 % across visible range, TiO_2_—known for its broadband high reflectance—was introduced as a dopant. Increasing the TiO₂ weight ratio (*i.e.,* *wt%*) enhances NIR reflectance and maintains over 90% reflectance in the visible range above 0.41 μm. Nonetheless, the optical characteristics of TiO_2_ inherently reduce reflectance in the violet region (*i.e.,* 0.38–0.41 μm), leading to a shift in the material’s color appearance. b) Chromatic characteristic of white-color pigments in the color space. The chromatic analysis based on the reflectance spectrum of the Al_2_O_3_ and 1 *wt%* of TiO_2_ samples revealed that their color coordinates (X, Y) closely matched the sRGB-defined white region.

**
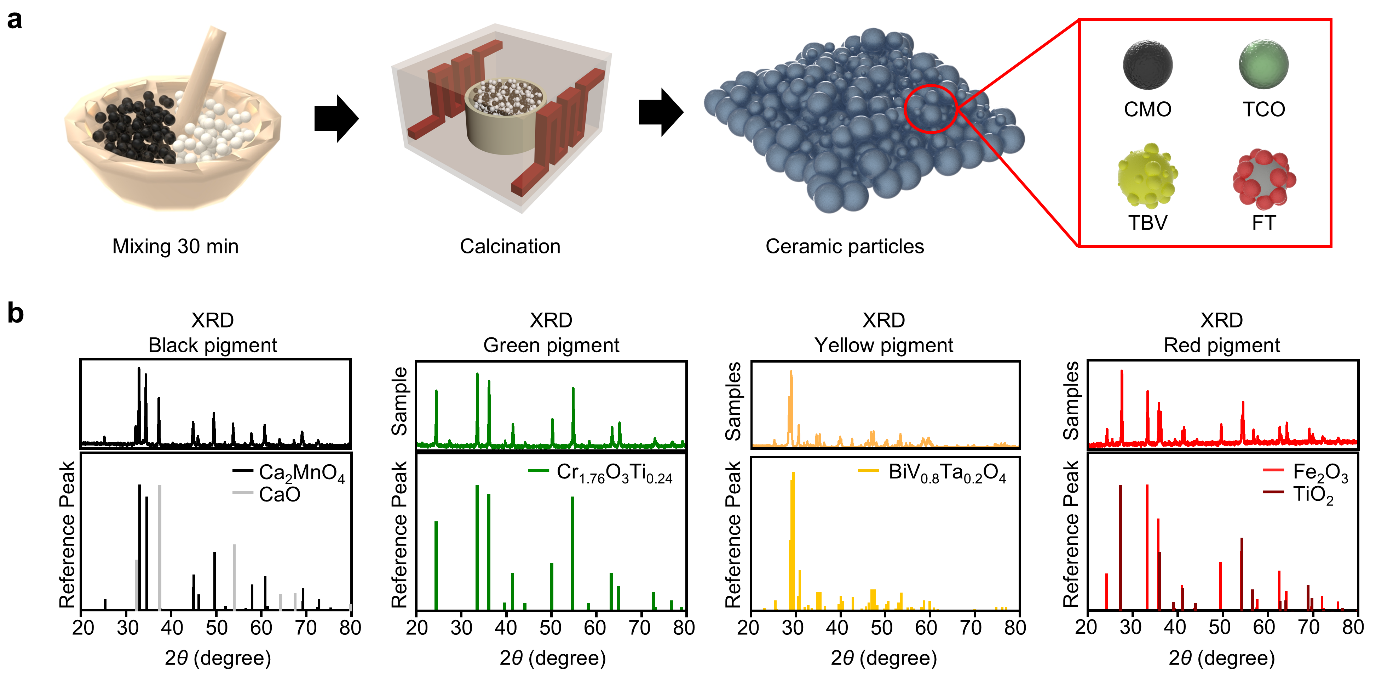
**

**Figure S3.** Fabrication of bi-function ceramic pigment (BFCP). a) Illustration of BFCP powder process. To further improve NIR reflectance, ceramic materials of various colors are doped with titanium (Ti), manganese (Mn), and tantalum (Ta) ions. These are fabricated by mixing materials as a specific mass ratio and calcinating them at the optimal temperature for each material. The result of fabrication is the BFCP with diverse color (*e.g.,* Black, Green, Yellow, and Red). b) Analysis of BFCP components. X-ray diffraction (XRD) characterization of BFCP component illustrates that BFCP_black_ is a Mn-doped CaCO_3_ (CMO), BFCP_green_ is a Ti-doped Cr_2_O_3_ (TCO), BFCP_yellow_ is a Ta-doped BiVO_4_ (TBV), and BFCP_red_ is consists of Fe_2_O_3_ and TiO_2_ (FT).

**
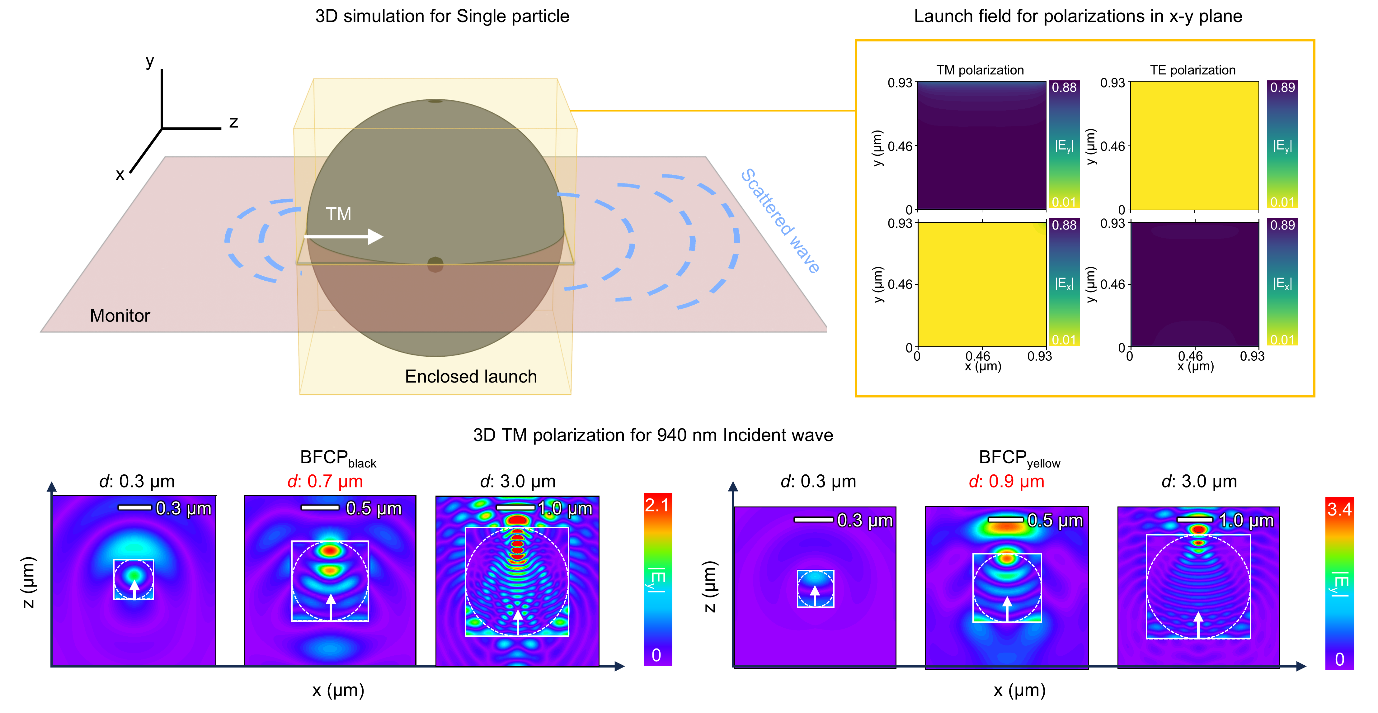
**

**Figure S4.** Optical simulation of single particle. Based on the finite difference time-domain (FDTD), we can examine the scattered wave by a single particle. In 3D simulation, the launch field is a cube, and polarized light (*i.e.,* TM and TE) propagates along the z-axis within the x-y plane from one of its six faces. Inside the cube, both scattered and non-scattered waves interact with the particle. Upon exiting the launch field, only the scattered waves propagate outward, while the non-scattered waves are absorbed and dissipate. Consequently, scattered waves can be observed in the external region. For example, as result of 3D simulation, cross-sectional electric field map of monitor illustrates that as predicted by the calculation in Supplementary Figure S1 and Figure 2a, the backscattered wave intensity is maximized under Mie scattering conditions.

**
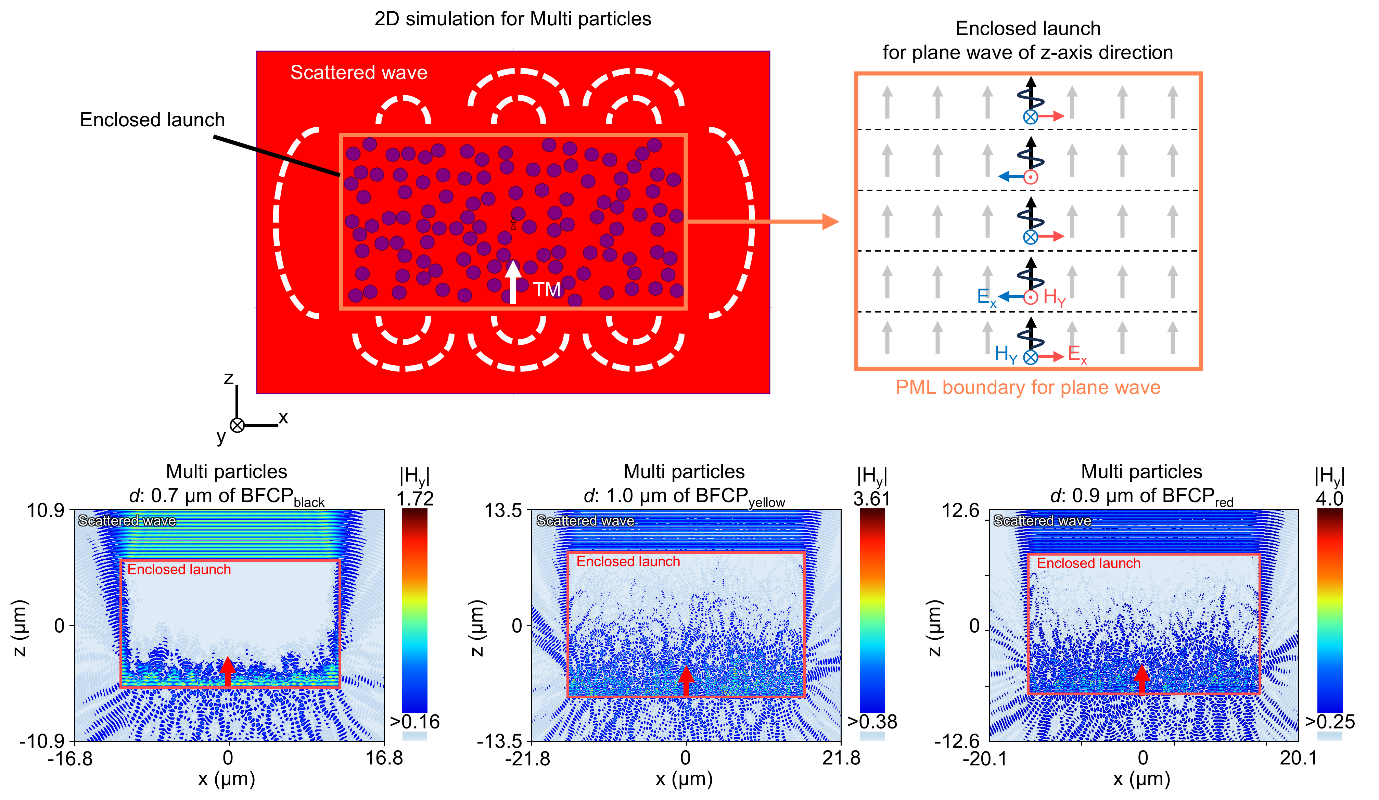
**

**Figure S5.** Optical simulation of multiple particles. Based on the finite difference time-domain (FDTD), due to the excessive computational time and data requirements, a 3D simulation with multiple particles becomes unmanageable, necessitating the use of a 2D simulation. In 2D simulation, the launch field is a square (*i.e.,* x-z plane), and polarized light (*i.e.,* TM and TE) propagates along the z-axis. In 2D simulation, circles are interpreted as infinitely extended cylinders along the y-axis rather than spheres. Therefore, TM polarization, where the electric field (E_x_) propagates parallel to the z-x plane, was used instead of TE polarization. Inside the launch field, both scattered and non-scattered waves interact with multiple particles. Upon exiting the launch field, only the scattered waves propagate outward, while the non-scattered waves are absorbed and dissipate. Consequently, scattered waves can be observed in the external region. For example, as result of 2D simulation, cross-sectional magnetic field map of monitor illustrates that Mie-scale particles lead to a substantial increase in effective backscattered wave distribution.

**
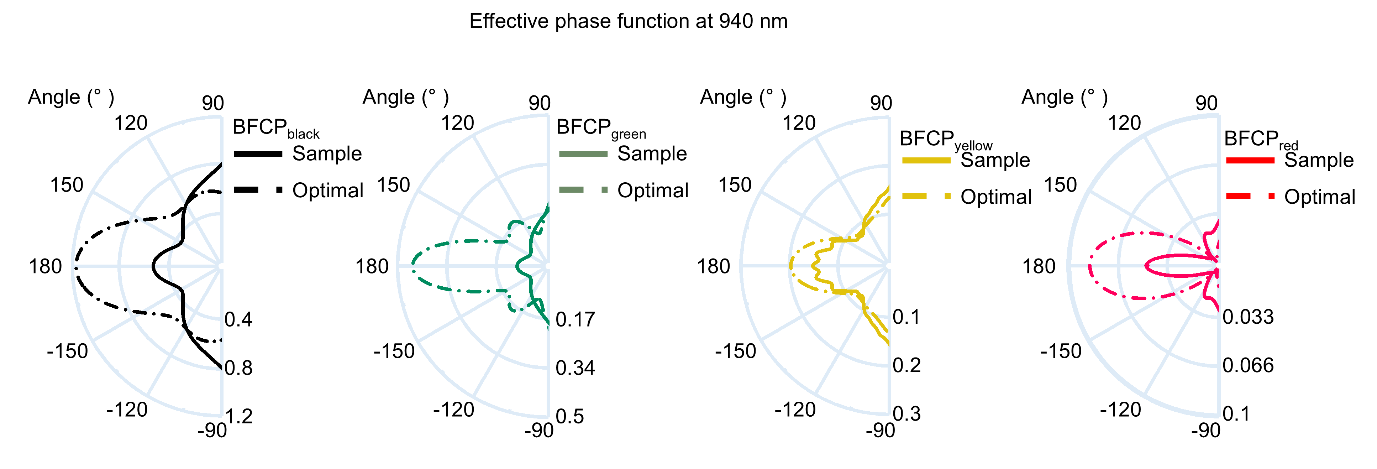
**

**Figure S6.** Comparison of the effective phase function between optimal BFCP and BFCP sample.

**
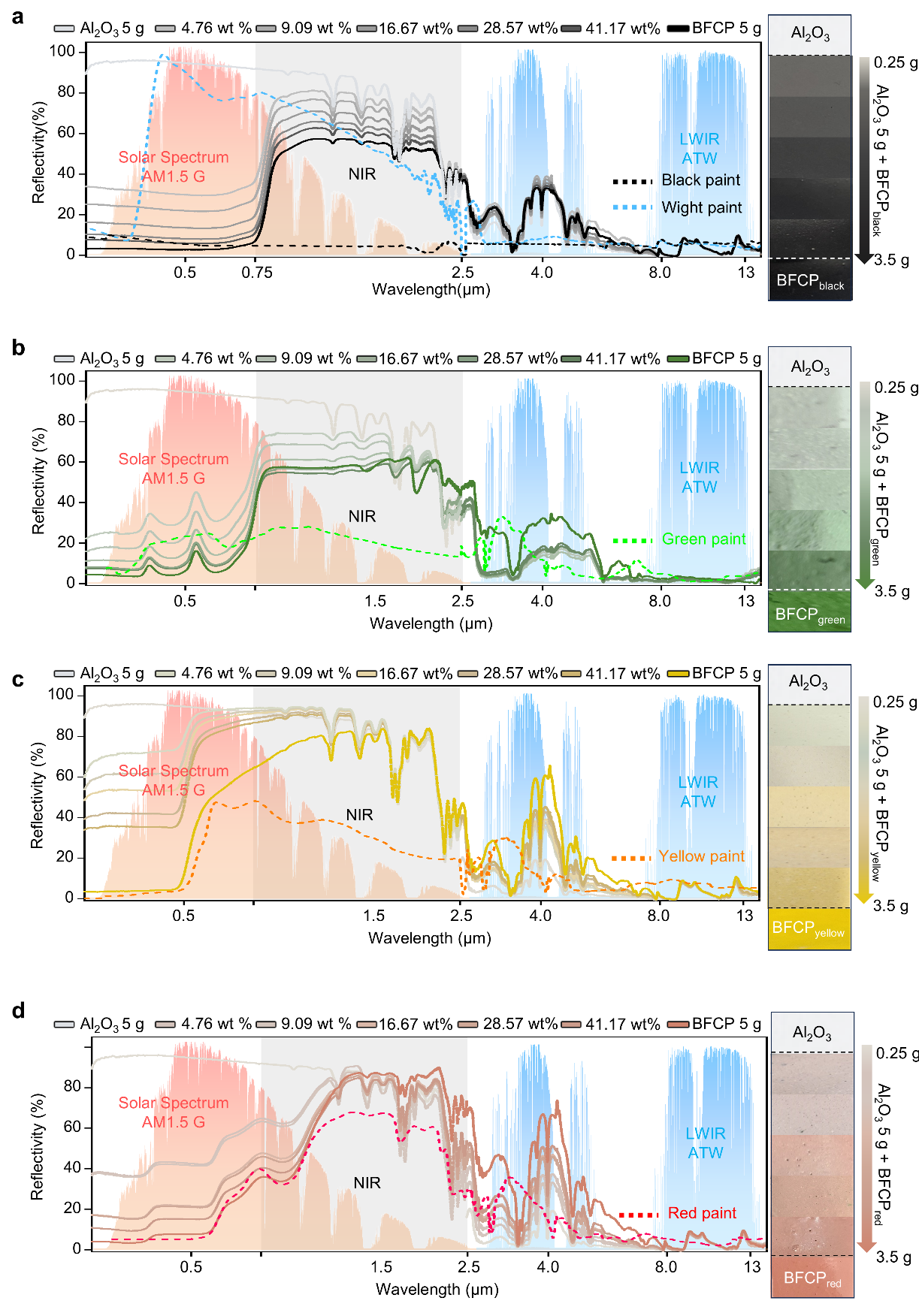
**

**Figure S7.** Optical properties of BFCP. For the weight ratio of BFCP with white pigment (Al_2_O_3_) (*i.e.,* wt%: BFCP/(BFCP+Al_2_O_3_)), the optical properties were measured using spectrophotometer in visible range (*e.g.,* 0.4–2.5 μm), and a FT-IR spectrometer in IR spectrum (*e.g.,* 2.5–16.6 μm). a) Graph of the optical characteristics of BFCP_black_. b) Graph of the optical characteristics of BFCP_green_. c) Graph of the optical characteristics of BFCP_yellow_. d) Graph of the optical characteristics of BFCP_red_.

**
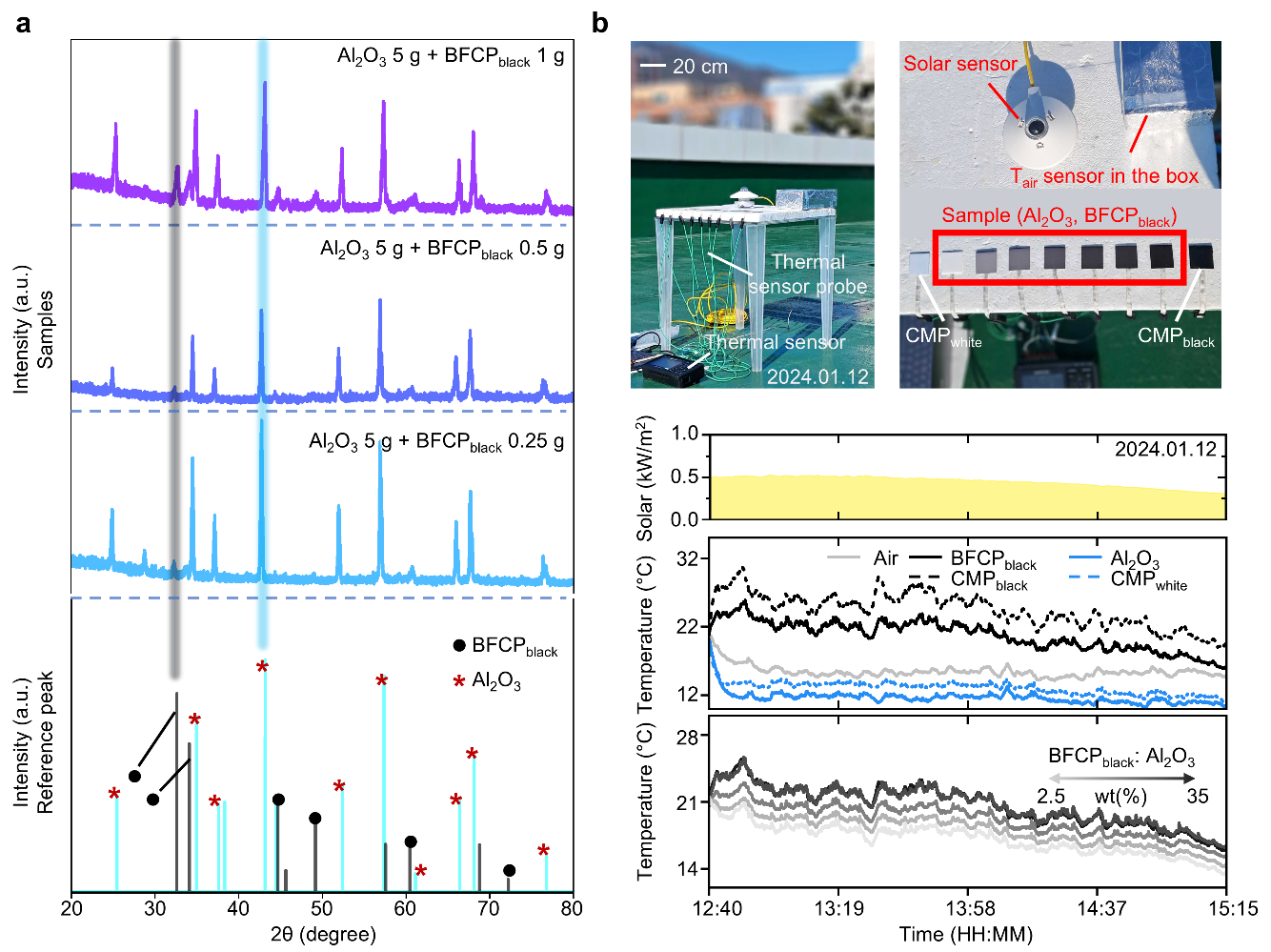
**

**Figure S8.** Outdoor cooling experiment as a function of weight ratio (wt%). a) X-ray diffraction (XRD) characterization of BFCP component as a function of wt%. For the weight ratio of BFCP_black_ with white pigment (Al_2_O_3_) (*i.e.,* wt%: BFCP/(BFCP + Al_2_O_3_)), an increase in wt% leads to a more pronounced influence of BFCP. b) Outdoor cooling experiment. The temperature was measured by placing samples sequentially from left to right, including white pigment (Al_2_O_3_), varying wt% compositions (*i.e.,* 2.5–35 %), and BFCP_black_ (*i.e.,* 100 %), along with CMP_black_ and CMP_white_. As a result, it was confirmed that the cooling effect varies depending on wt%, and the difference in cooling performance between CMP and BFCP was also observed.

**
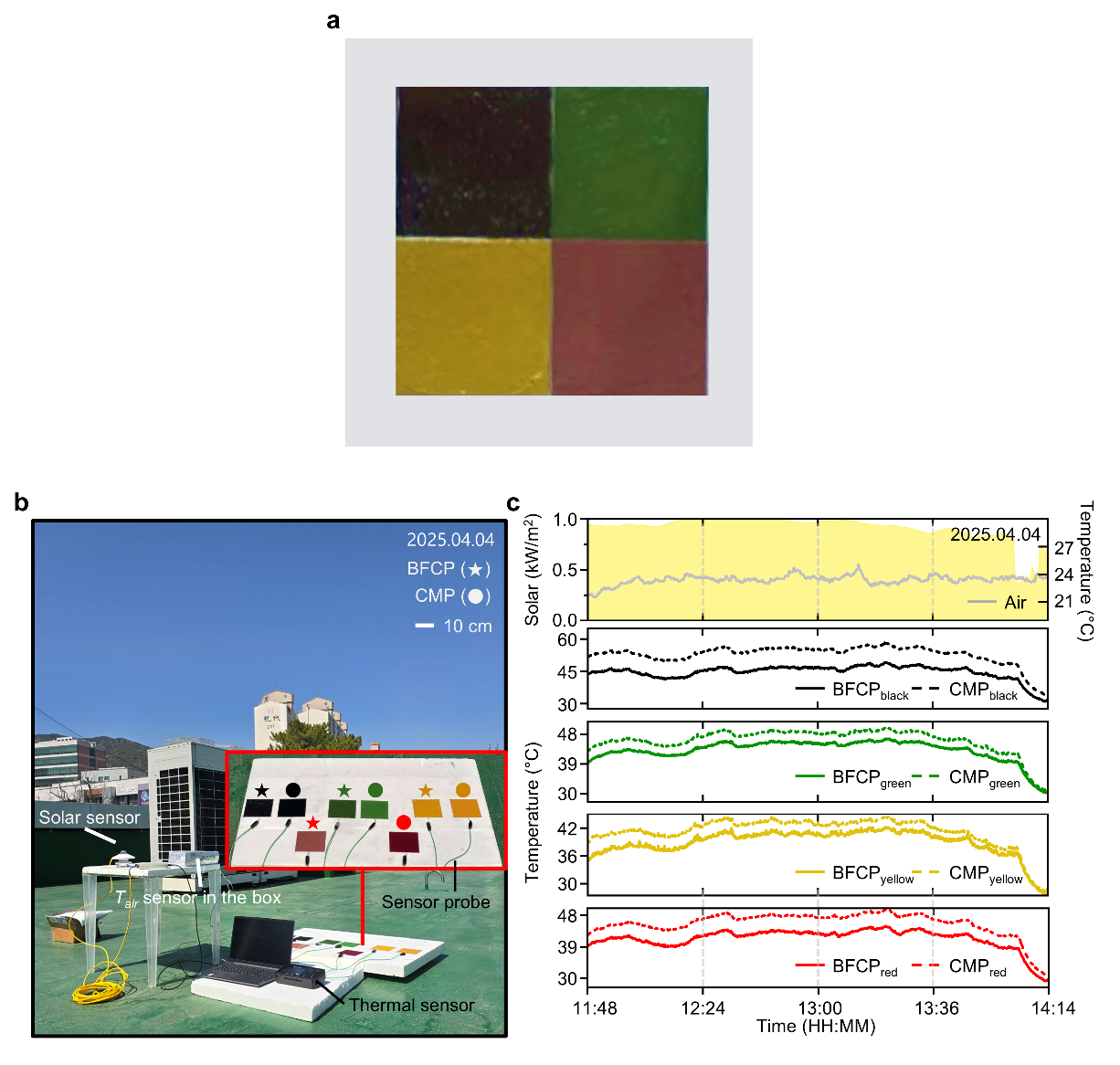
**

**Figure S9.** BFCP coating on steel sheet. a) Image of BFCP coating on the steel sheet. b) Configuration of the thermal experiment. c) Outdoor temperature measurements of the steel sheets coated with BFCP and CMP.

**
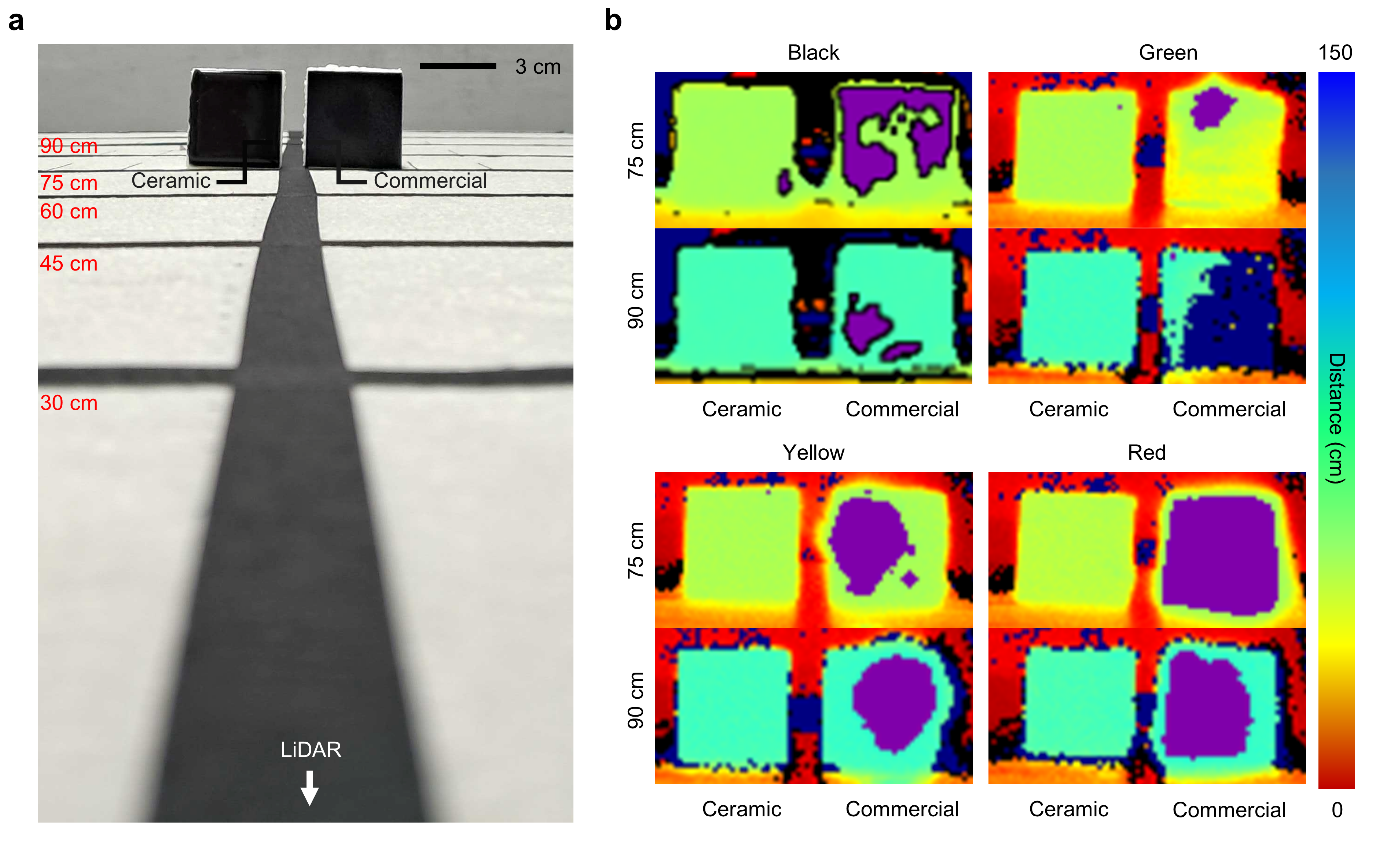
**

**Figure S10.** Illustration of LiDAR experiment setup and LiDAR detection outcomes relative to the measurement length (*L*). a) Image of the LiDAR experiment setup. b) LiDAR detection experiment results. In the LiDAR detection range (*e.g.,* 0–150 cm), by placing BFCP and CMP at the same distance and simultaneously detecting them with a LiDAR sensor, the LiDAR detection capability of BFCP can be evaluated.

**
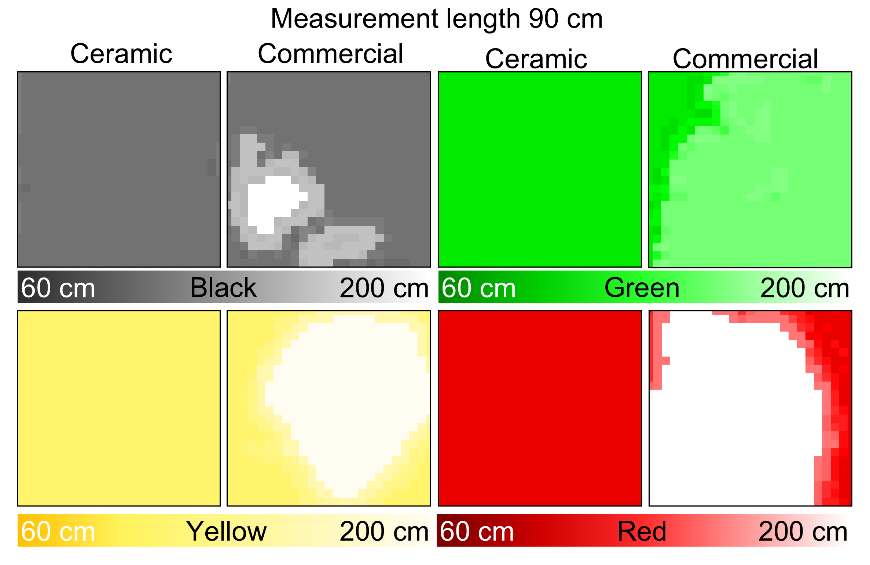
**

**Figure S11.** Mapping process from detected data at measurement length of 90 cm.

**
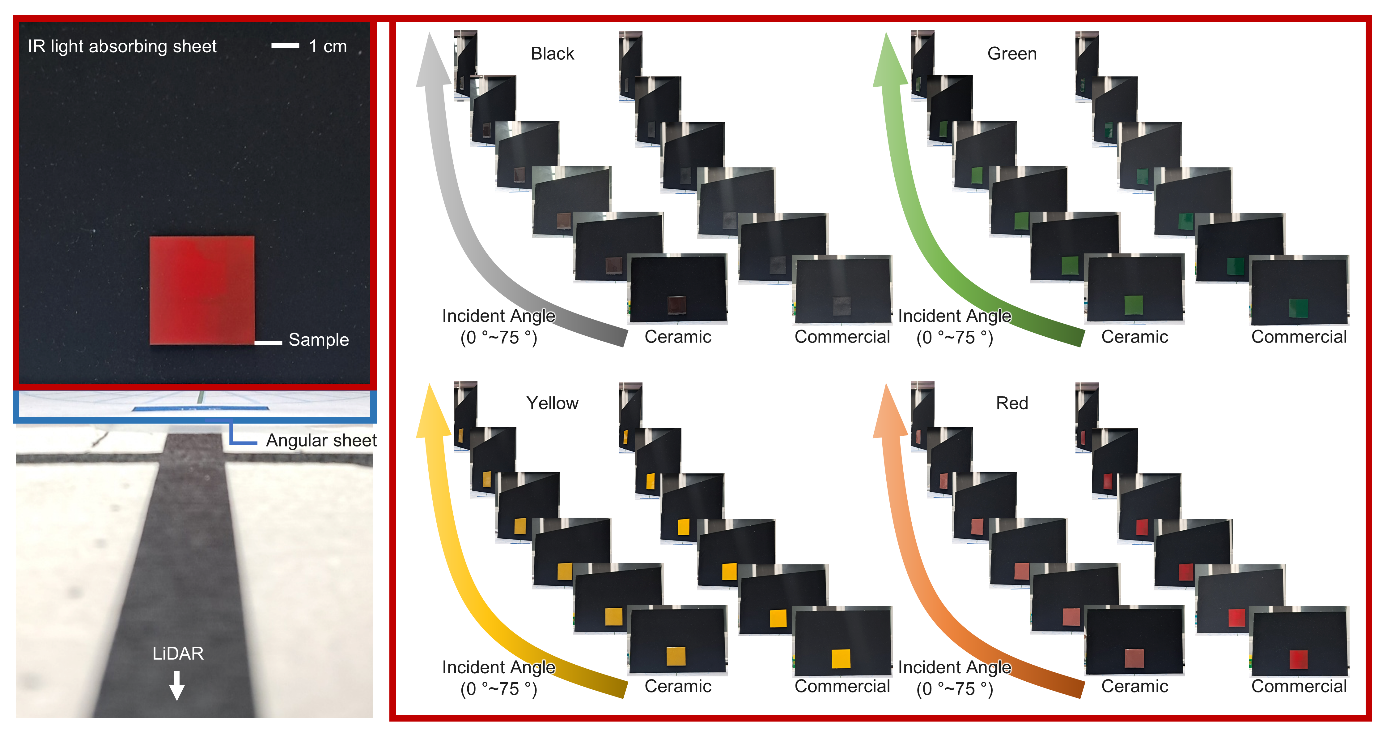
**

**Figure S12.** Schematic of the LiDAR experiment setup as a function of the incident angle.


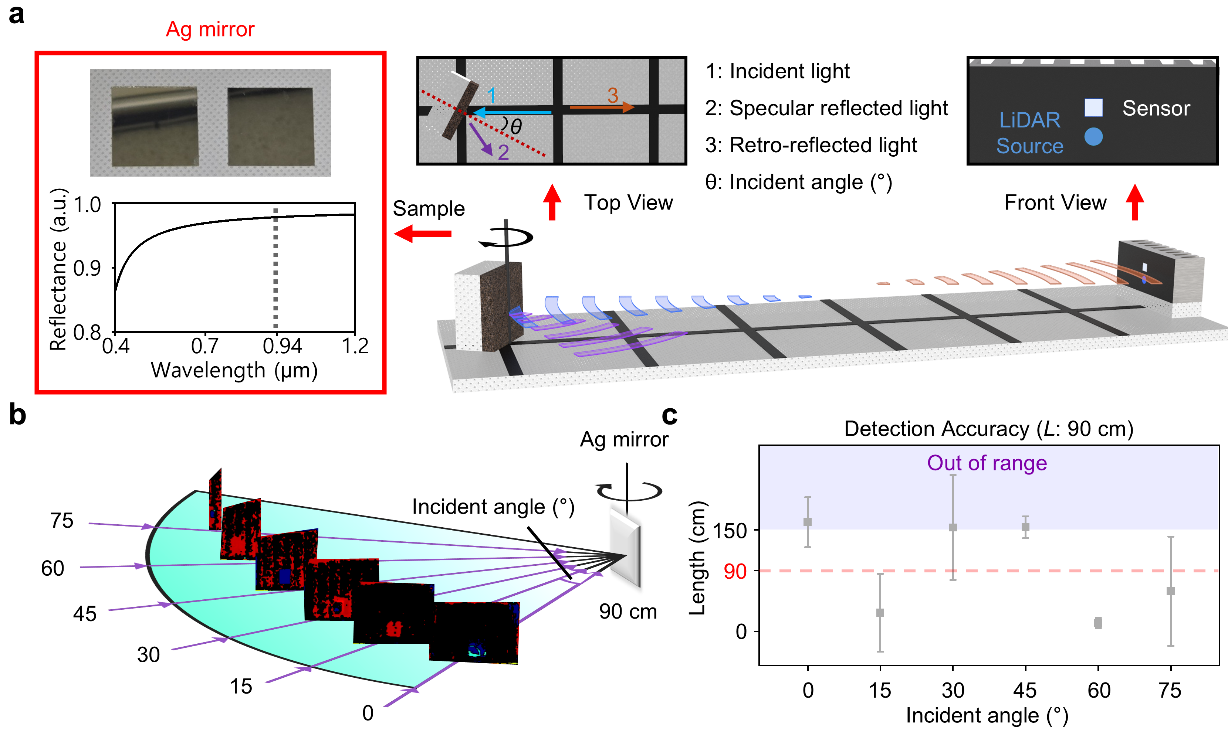


**Figure S13.** The Inner LiDAR test involving the Ag mirror. a) The Ag mirror exhibits near unity reflectance at the LiDAR wavelength (*i.e.,* 0.94 μm). However, due to the highly polished surface, the Ag mirror primarily induces specular reflection rather than scattering. b) Illustration of LiDAR detection outcomes relative to the incident angle for Ag mirror. c) Graph of detection accuracy. In the LiDAR detection range (*e.g.,* 0–150 cm), the results indicate that although the Ag mirror exhibits high reflectance, the Ag mirror is not effectively detected due to dominant specular reflection. In addition, at an incident angle of 0 °, the mirror was not properly aligned with the sensor, resulting in a failure of LiDAR detection.


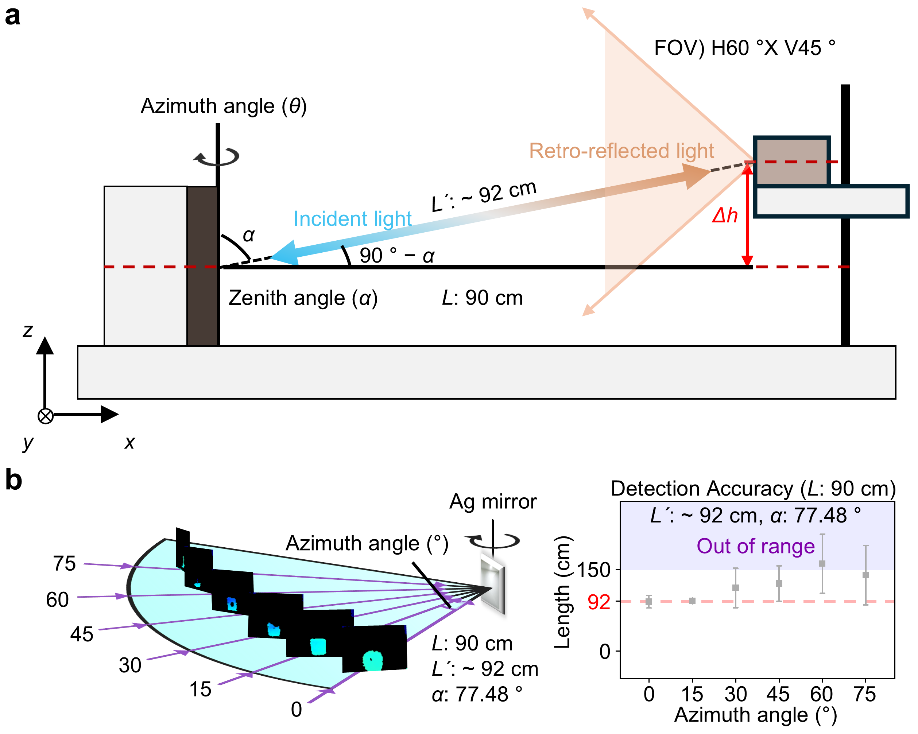


**Figure S14.** The Inner LiDAR test under a spherical coordinate. a) Configuration of the LiDAR experiment. The height (*i.e.,* *Δh*) of the LiDAR sensor is varied to define angular variables in a spherical coordinate system. When the *Δh* was set to 20 cm, although the horizontal distance (*i.e., L*) was 90 cm, the actual distance (*i.e., L΄*) between the sensor and the sample was approximately 92 cm, corresponding to a zenith angle (*i.e.,* $\alpha$) of ~77.48 °. b) Illustration of LiDAR detection outcomes relative to the Azimuth angle for Ag mirror. Measurements were more susceptible to environmental influences compared to the previous experiments (Figure S 13). In particular, the white surface of the floor in the experimental setup occasionally caused LiDAR detection, even when the signal was not directly reflected from the sample.


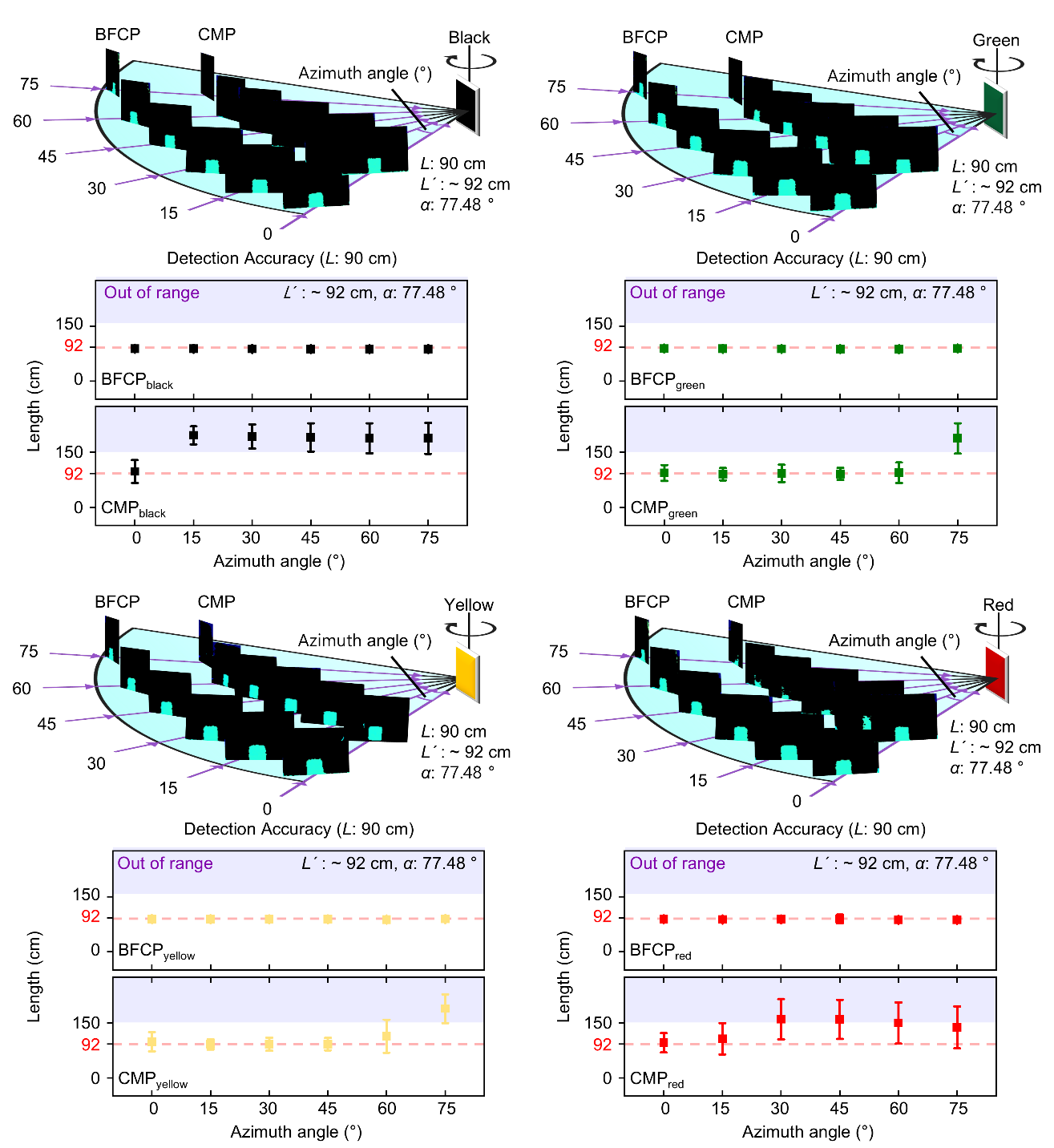


**Figure S15.** LiDAR detection performance of BFCP and CMP as a function of azimuth angle. The BFCP sample demonstrated consistent LiDAR detectability regardless of color or angles. In contrast, the CMP sample was significantly affected by the surrounding environment, leading to inconsistent detection and reduced performance at higher azimuth angles.


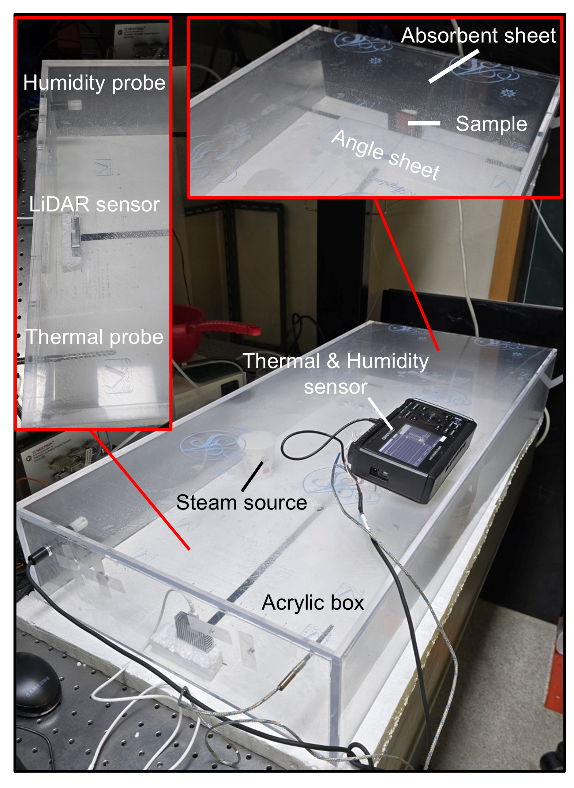


**Figure S16.** Configuration of the LiDAR experiment as a function of humidity. The existing setup was enclosed within an acrylic box to create a controlled environment. A steam source was introduced to regulate the internal temperature and humidity, while thermal and humidity sensors were used to monitor ambient conditions. This experiment was conducted at a temperature of 24–25 °C and a relative humidity of approximately 90–91 %. To ensure more accurate assessment of the detection performance, an absorbent sheet was placed behind it to improve contrast. In addition, an angled sheet was employed to rotate the sample, allowing precise control of the incident angle.


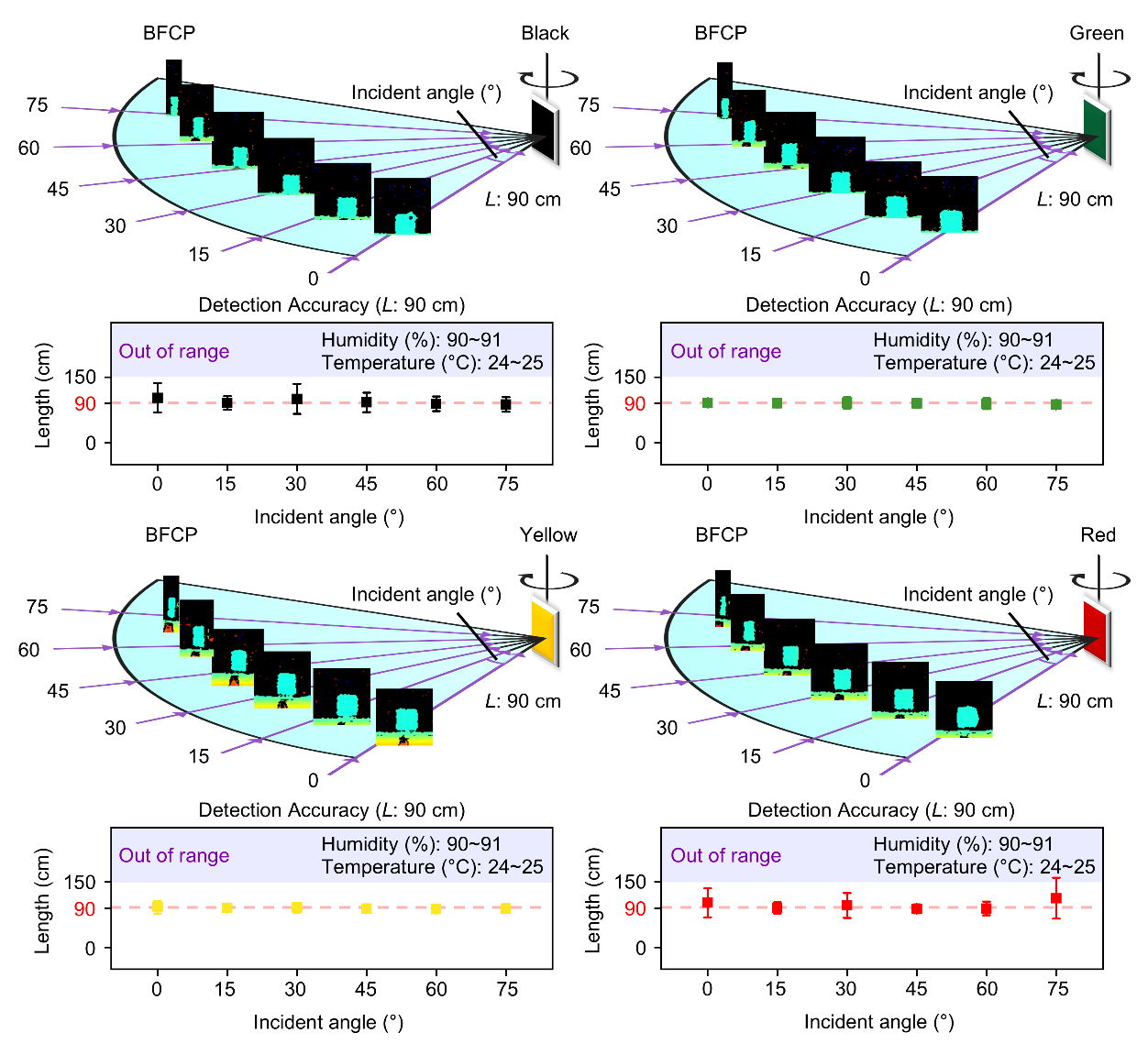


**Figure S17.** Illustration of LiDAR detection results for BFCP with respect to humidity. While slight variations were observed compared to prior experiments as shown in Figure S15 due to the influence of water vapor, the sample was still consistently detected at comparable distances. More importantly, the results confirm that the sample is reliably recognized by the LiDAR system regardless of the incident angle.

**
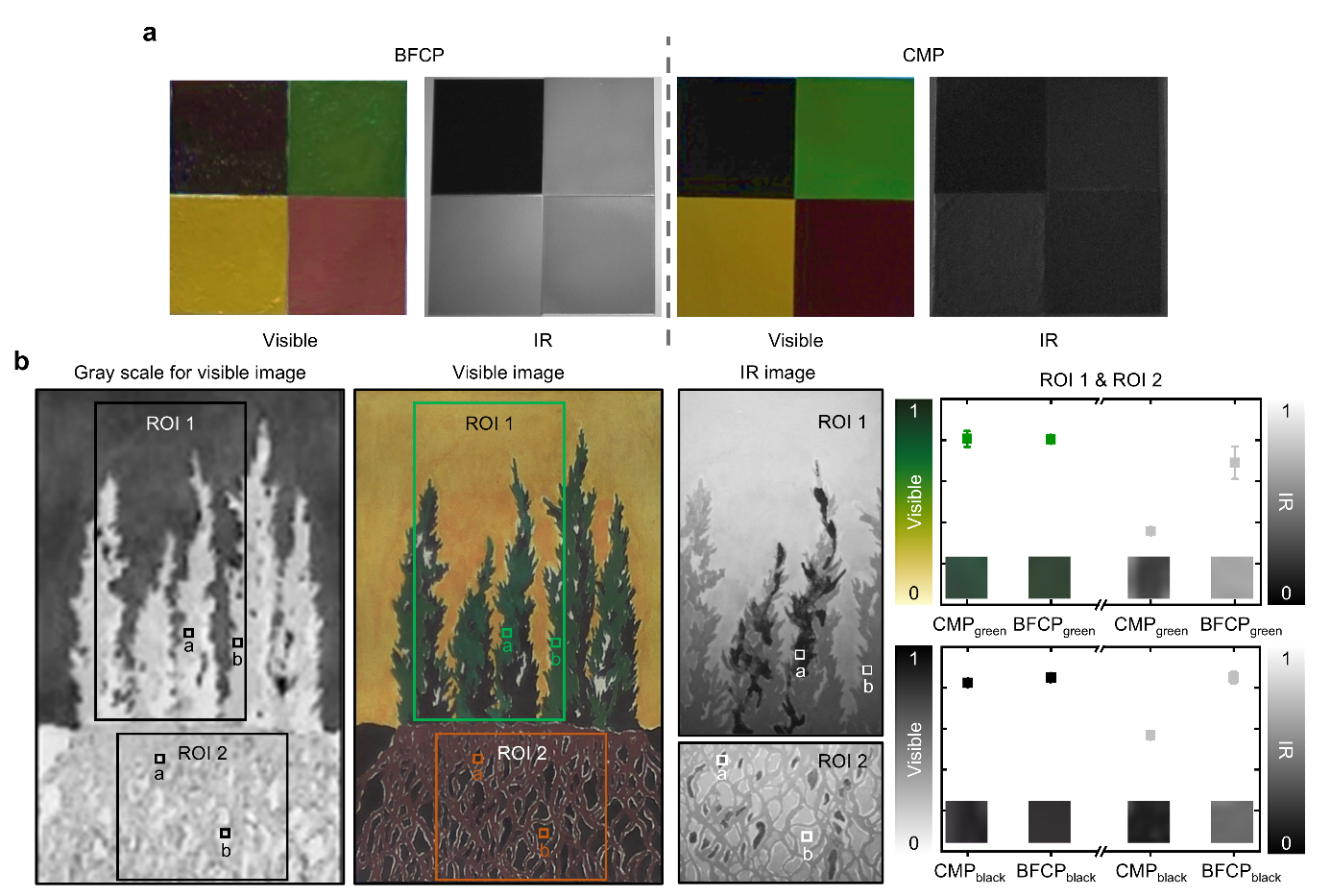
**

**Figure S18.** Anti-counterfeiting application of BFCP. a) Images of BFCP and CMP coating on the steel sheet. b) Anti-counterfeiting painting utilizing BFCP paint. BFCP powder can be easily converted into paint by mixing it with a glue solution (Sukinikawa-eki, NAKAGAWA GOFUN ENOGU, Japan). Due to its high NIR reflectance, it remains visually indistinguishable when combined with commercial paints (CMP). However, when observed using an IR camera (HEXEUM, United States of America), the difference in reflectance enables the creation of painting that can conceal specific messages. For example, In the visible image and its corresponding gray scale representation, the region of interest (ROI) consists of CMP paint (a) and BFCP (b). Both ROI 1 and ROI 2 remain non-sensing in the visible image. However, due to the intensity difference BFCP paint and CMP, additional features are revealed in ROI 1 and ROI 2 within the IR image.


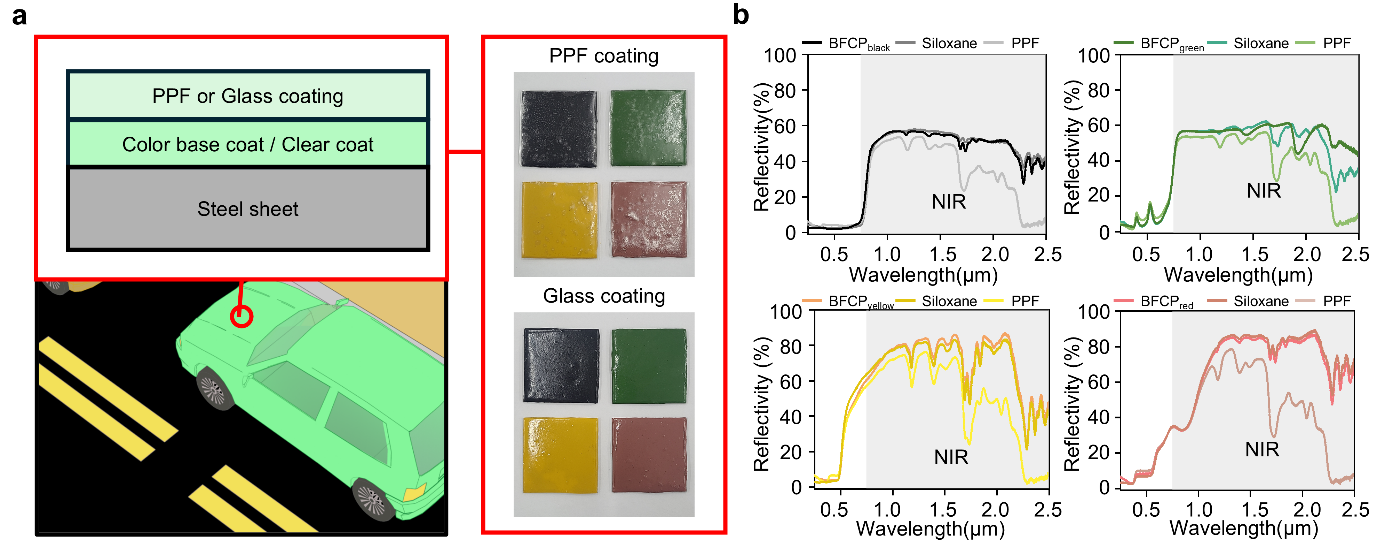


**Figure S19.** Multiple coating layers of automotive painting procedure. a) Schematic of the automotive coating structure. The process of coating begins with the application of a color base coat and a clear coat to achieve a clean and uniform appearance. Subsequently, an additional protective layer, such as paint protection film (PPF) or glass coating, is often applied to shield the surface from external environmental factors. b) Graph of the optical characteristics of coating structures. The glass coating had a minimal impact on the optical properties, as no significant difference was observed between the coated and uncoated samples. In contrast, the PPF-coated sample exhibited a slight deviation in optical properties beyond 1.5 μm. However, the solar spectral intensity is relatively low in this range and commercial LiDAR typically operate within 0.9–1.5 μm. Therefore, this difference is not expected to compromise the intended radiative cooling and LiDAR sensing functionality of the coating in autonomous vehicle applications.


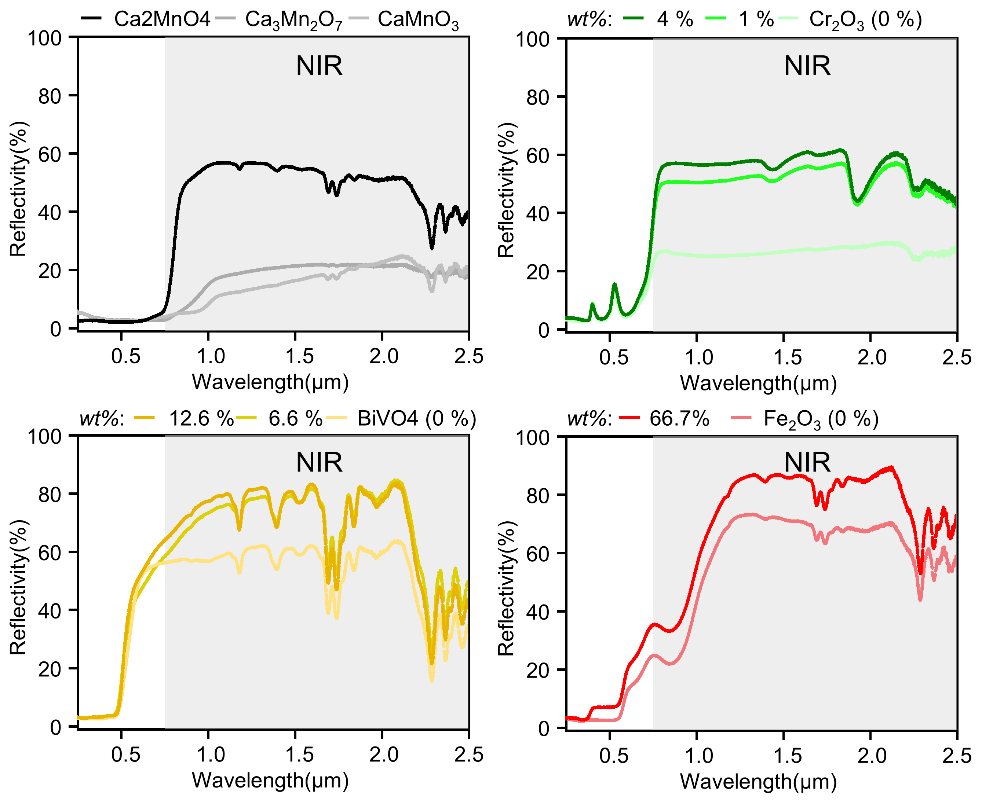


**Figure S20.** Optical properties of color-specific BFCP samples depending on the weight ratio (*i.e.,* *wt%*).

**Tables**

**
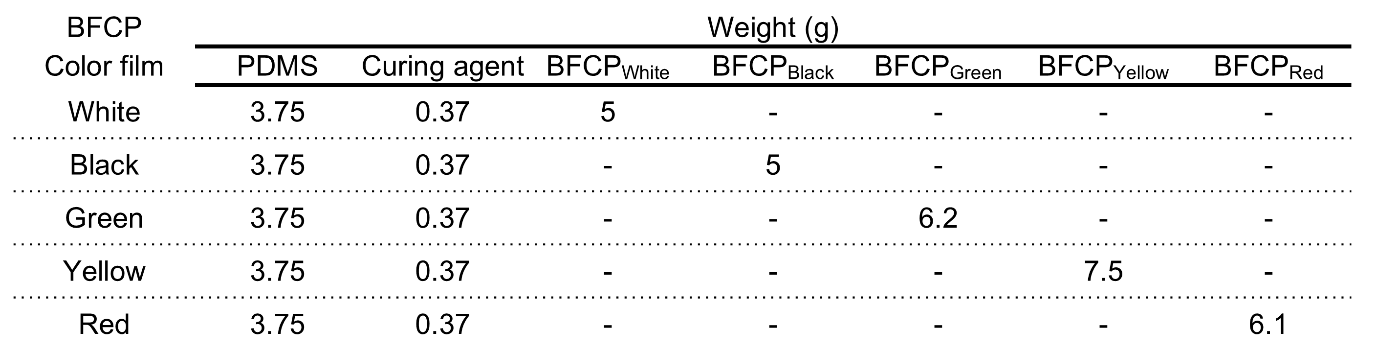
**

**Table S1.** Fabrication ratio of BFCP color film.

**
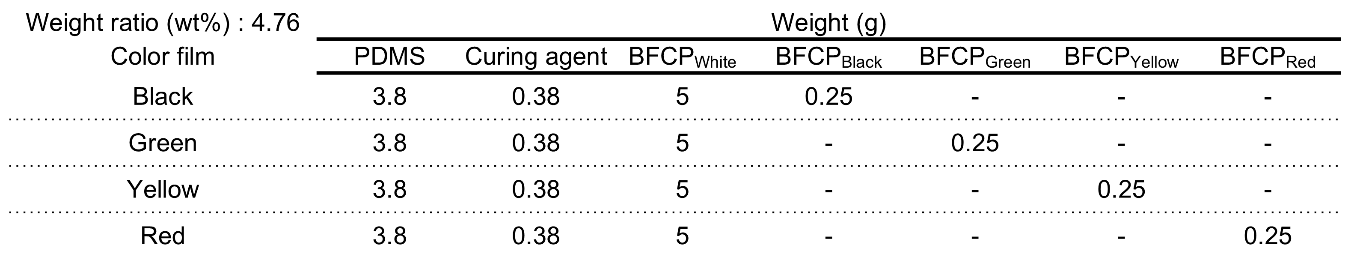
**

**Table S2.** Fabrication ratio of BFCP color film with a 4.76 % BFCP composition (*i.e.,* wt%: BFCP/(BFCP+Al_2_O_3_)).

**
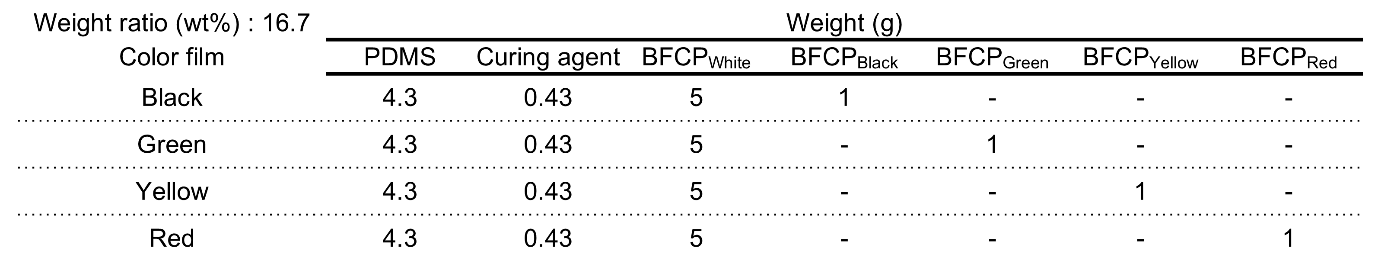
**

**Table S3.** Fabrication ratio of BFCP color film with a 16.7 % BFCP composition (*i.e.,* wt%: BFCP/(BFCP+Al_2_O_3_)).


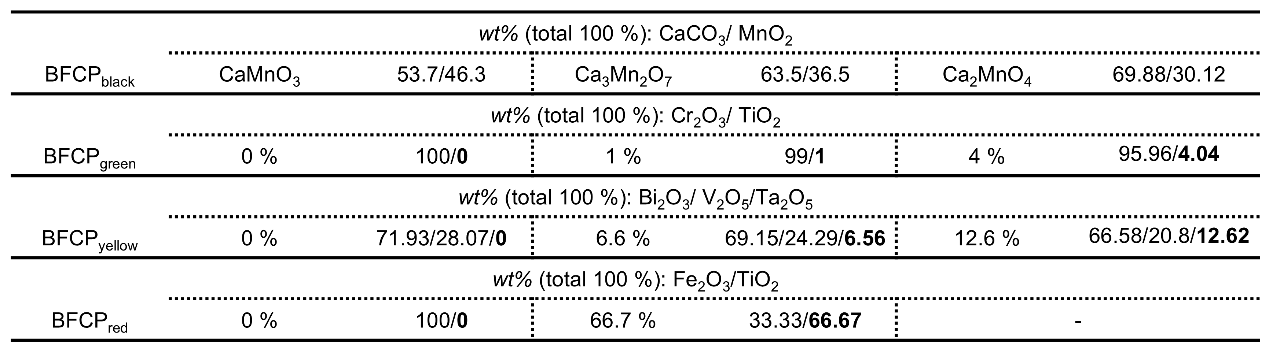


**Table S4.** Weight ratios (*i.e., wt%*) for color-specific BFCP samples.

**Reference**

[1] R. Oka, T. Masui, *RSC Advances* **2016**, *6*, 90952.

[2] Y. Zhou, X. Weng, L. Yuan, L. Deng, *Journal of the Ceramic Society of Japan* **2014**, *122*, 311.

[3] L. Sandhya Kumari, P. Prabhakar Rao, A. Narayana Pillai Radhakrishnan, V. James, S. Sameera, P. Koshy, *Solar Energy Materials and Solar Cells* **2013**, *112*, 134.

[4] H. J. Lee, K. Y. Jung, Y.-S. Kim, *RSC Advances* **2021**, *11*, 16834.

[5] S. Wolf, N. V. Voshchinnikov, *Computer Physics Communications* **2004**, *162*, 113.

[6] Z. Huang, X. Ruan, *International Journal of Heat and Mass Transfer* **2017**, *104*, 890.

[7] J. Peoples, X. Li, Y. Lv, J. Qiu, Z. Huang, X. Ruan, *International Journal of Heat and Mass Transfer* **2019**, *131*, 487.

[8] W. J. Wiscombe, *Applied Optics* **1980**, *19*, 1505.

[9] C. F. Bohren, D. R. Huffman, **1998**, DOI https://doi.org/10.1002/9783527618156.

[10] Matzler, Christian. (2002). MATLAB functions for Mie scattering and absorption. IAP Res Rep. 8.
